# A whole slide image-based machine learning approach to predict ductal carcinoma in situ (DCIS) recurrence risk

**DOI:** 10.1186/s13058-019-1165-5

**Published:** 2019-07-29

**Authors:** Sergey Klimov, Islam M. Miligy, Arkadiusz Gertych, Yi Jiang, Michael S. Toss, Padmashree Rida, Ian O. Ellis, Andrew Green, Uma Krishnamurti, Emad A. Rakha, Ritu Aneja

**Affiliations:** 1 Department of Biology, Georgia State University, Atlanta, GA 30303 USA; 2 Department of Cellular Pathology, University of Nottingham, Nottingham, UK; 3 Department of Pathology, Cedars-Sinai Medical Center, Los Angeles, CA USA; 4 Department of Mathematics and Statistics, Georgia State University, Atlanta, GA USA; 5 Department of Pathology, Emory University, Atlanta, GA USA; 6 Division of Cancer and Stem Cells School of Medicine, University of Nottingham City Hospital Campus, Nottingham, NG5 1PB UK

**Keywords:** DCIS, Digital image analysis, Prognosis, Machine learning, Recurrence prediction, Biomarker

## Abstract

**Background:**

Breast ductal carcinoma in situ (DCIS) represent approximately 20% of screen-detected breast cancers. The overall risk for DCIS patients treated with breast-conserving surgery stems almost exclusively from local recurrence. Although a mastectomy or adjuvant radiation can reduce recurrence risk, there are significant concerns regarding patient over-/under-treatment. Current clinicopathological markers are insufficient to accurately assess the recurrence risk. To address this issue, we developed a novel machine learning (ML) pipeline to predict risk of ipsilateral recurrence using digitized whole slide images (WSI) and clinicopathologic long-term outcome data from a retrospectively collected cohort of DCIS patients (*n* = 344) treated with lumpectomy at Nottingham University Hospital, UK.

**Methods:**

The cohort was split case-wise into training (*n* = 159, 31 with 10-year recurrence) and validation (*n* = 185, 26 with 10-year recurrence) sets. The sections from primary tumors were stained with H&E, then digitized and analyzed by the pipeline. In the first step, a classifier trained manually by pathologists was applied to digital slides to annotate the areas of stroma, normal/benign ducts, cancer ducts, dense lymphocyte region, and blood vessels. In the second step, a recurrence risk classifier was trained on eight select architectural and spatial organization tissue features from the annotated areas to predict recurrence risk.

**Results:**

The recurrence classifier significantly predicted the 10-year recurrence risk in the training [hazard ratio (HR) = 11.6; 95% confidence interval (CI) 5.3–25.3, accuracy (Acc) = 0.87, sensitivity (Sn) = 0.71, and specificity (Sp) = 0.91] and independent validation [HR = 6.39 (95% CI 3.0–13.8), *p* < 0.0001;Acc = 0.85, Sn = 0.5, Sp = 0.91] cohorts. Despite the limitations of our cohorts, and in some cases inferior sensitivity performance, our tool showed superior accuracy, specificity, positive predictive value, concordance, and hazard ratios relative to tested clinicopathological variables in predicting recurrences (*p* < 0.0001). Furthermore, it significantly identified patients that might benefit from additional therapy (validation cohort *p* = 0.0006).

**Conclusions:**

Our machine learning-based model fills an unmet clinical need for accurately predicting the recurrence risk for lumpectomy-treated DCIS patients.

**Electronic supplementary material:**

The online version of this article (10.1186/s13058-019-1165-5) contains supplementary material, which is available to authorized users.

## Introduction

The incidence of ductal carcinoma in situ (DCIS) has rapidly risen over the past few decades [1] and is estimated to affect over 1 million US women by 2020 [2]. Despite the excellent overall survival of DCIS patients [3, 4], over-treatment is a considerable concern [5], which results mainly from the inability of standard clinicopathologic factors to accurately identify a low-risk group unlikely to recur [6, 7].

One of the goals of DCIS treatment is to curb local recurrence, especially invasive recurrence. Common histopathological factors such as age at diagnosis, DCIS growth pattern, tumor size, margin status, nuclear grade, presence of comedo necrosis [8, 9], and combinations of the aforementioned (such as in the Van Nuys Prognostic Index or in prognostic nomograms) [10, 11] have been shown to have limited value in predicting recurrence. Efforts to introduce new DCIS molecular prognostic variables have not offered consistent results [12] nor were they found to be significantly prognostic tools [13]. Additionally, transcriptomic models have restrictive requirements [14], are not cost-effective [15], lack significant “genetic patterns leading to invasive disease” signatures [7], and do not take into account the tumor stromal microenvironment. Thus, there is an unmet clinical need for novel tools to improve recurrence risk stratification of DCIS [16].

With the advent of technology able to process data in a high-throughput manner, computational pathology has shown promise as a valuable prognostic tool. By integrating image analysis, data generation, and medical statistics, computational pathology enables a high-level quantitative tissue analysis [17, 18]. Although relatively new, computational pathology has already shown marked success in assisting with diagnosis, tumor classification, and predicting patient prognosis in a variety of cancer types [19–24]. Whole slide quantitative image analysis pipelines have demonstrated significant discriminatory success not only using features stemming from pixel (stain) intensities [25, 26], but also morphometric features and texture [27, 28]. For predicting DCIS recurrence, various scales of these image features have been studied using H&E-stained tissue, such as through quantifying image features of comedo necrosis within ducts [29]. At the cellular level, chromatin distribution, long considered a computationally quantifiable feature of cancer cells [30], has also been used to predict DCIS recurrence [31, 32] and was shown to outperform its pathological analog, nuclear grade [33]. However, these results focus on a narrow range of very specific characteristics of the DCIS and discard the rich information that could potentially be derived from consideration of other architectural features (e.g., surrounding stromal, blood vessel-related) within the sample.

Human eye limitations and lack of concordance between pathologists impact DCIS grading in clinical practice. Notably, the breadth of DCIS grading is limited to a single (high-grade) duct, and oftentimes, histopathologic features are grouped into qualitative categories instead of capturing and analyzing more granular data derived from quantitative features. This simplification overlooks (a) the prognostic value of the surrounding microenvironment [34–36] and even alterations in non-cancerous epithelial cells [37] and (b) the tremendous intra-tumor heterogeneity, which cannot be categorized in a fundamentally meaningful way [38]. Our current study evaluates whether quantitatively analyzing the whole slide, dubbed whole slide image (WSI) analysis [39] has prognostic and predictive value with respect to the recurrence prediction for DCIS.

**Fig. 1:**
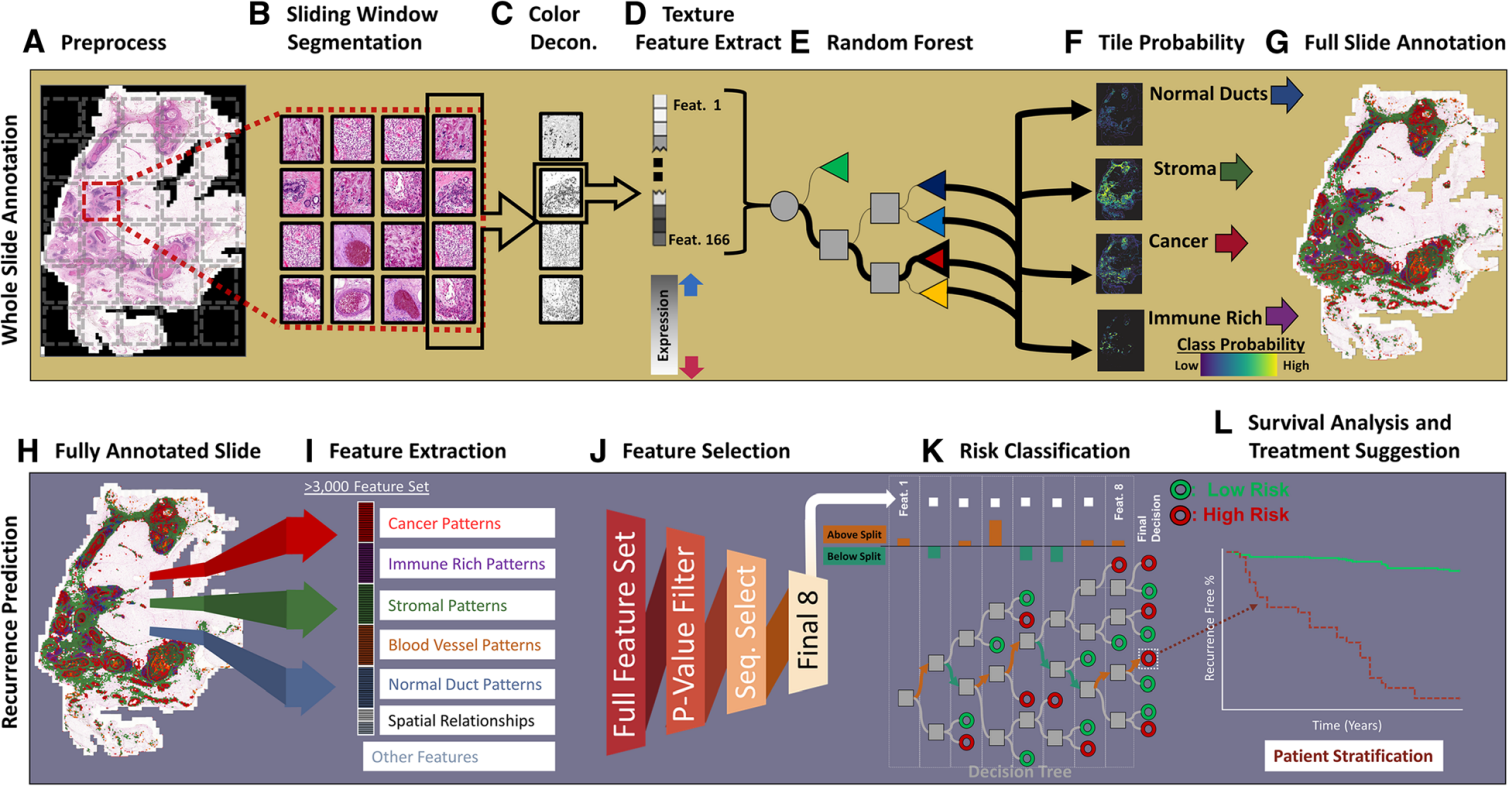
WSI method for stratifying DCIS patients based on their recurrence risk. The first step in this pipeline automatically annotates the patient’s whole surgical H&E slides into prognostically informative tissue classes. For this automated annotation, the patient’s whole virtual slide is **(a)** preprocessed through whole-slide color normalization and down-sampling followed by **(b)** a sliding window, over the whole slide, which extracts non-overlapping image tiles which are then **(c)** color deconvoluted to yield the hematoxylin image from which **(d)** values for 166 texture features are extracted. These features are then **(e)** input into a random forest annotation classifier which **(f)** outputs a probability of each tile belonging to a specific class (malignant ducts of DCIS, surrounding the breast parenchyma/ducts, blood vessels, and stromal regions with and without dense immune infiltration [immune cells occupying at least 50% of the tile area]) which are combined to produce **(g)** a whole-slide annotation. The second step extracts tissue architecture features and features of the spatial relationship between these tissue classes, from the previously annotated slides, and compiles them into what serves as the “full-slide” feature set. For the prediction of DCIS recurrence risk, **(h)** each annotation is analyzed through **(i)** feature distributions, spatial features which compare distances between different classes, and other features such as region confidence. **(j)** The final (optimized) feature list, alongside the patient’s follow-up (recurrence) data as the labels, is used to train a **(k)** random forest recurrence risk classifier to predict **(l)** high versus low risk of recurrence and allows for the recommendation of optimal therapy

In the retrospective study presented herein, we developed a machine learning-based image analysis pipeline, identified prognostically relevant features obtained from the texture of H&E slides [40], and designed a novel classification approach to predict 10-year recurrence risk in DCIS patients treated with breast-conserving surgery (BCS) (Fig. 1). Finally, to validate the prognostic value of this approach, and investigate its generalizability, the model was tested on a cohort of high-grade-only patients, traditionally seen as a high-risk group for recurrence [41].

## Methods

### Study population

The study population was obtained from patients diagnosed at Nottingham City Hospital (DCIS case series), spanning the period from 1989 to 2012. The training cohort comprised slides from 159 patients (127 of whom had multiple tumor blocks yielding a total of 335 slides); these slides were used for the model development (Table 1) and training. A further 185 patients (9 of whom had multiple slides, yielding a total of 199 slides) comprised an independent validation cohort for the recurrence risk classifier (Table 1). Patients included in this study were exclusively those presenting with pure DCIS (without any invasive component/tumor in the primary biopsy whether ductal, lobular, or any special type), without bilateral disease, and treated with BCS, rather than mastectomy. The DCIS classification was initially identified through pathological records and further verified through a review of slides by 2 pathologists (IMM and MST). Details on clinicopathological variables including the size, tumor grade (classified according to the three-tier nuclear grading system [42]), comedo necrosis (defined as the presence of central acellular necrosis with nuclear debris), and final margins; demographic information; and follow-up data/recurrence status were retrospectively obtained from patient medical records and validated by pathologists (IMM and MST). Post-BCS, patients at Nottingham were screened once a year until their 5th year, after which they were followed up every 3 years. Recurrence-free survival (RFS) was calculated from the date of pathologic diagnosis until the first ipsilateral breast local recurrence or last follow-up. Local recurrence (either invasive or DCIS) was considered as an event. Cases with contralateral recurrences, or those who developed a second lower-grade tumor, were treated as censored at the time of development to avoid mixing the recurrences with new primaries.

**Table 1:** Patient characteristics

Clinicopathologic characteristics of patients in the training and validation cohorts			
Baseline characteristic	Training cohort (*N* = 159)	Validation cohort (*N* = 185)	Difference (*p* value)
Patient age			
Median age (range), years	57 (30–83)	59 (36–77)	0.30
Age < 50, *n* (%)	26 (16.3)	23 (12.4)	
Age ≥ 50, *n* (%)	133 (83.7)	162 (87.6)	
Menopausal status, *n* (%)			
Pre	31 (19.5)	29 (15.7)	0.35
Post	128 (80.5)	156 (84.3)	
Presentation, *n* (%)			
Screening	85 (53.5)	120 (64.9)	0.03
Symptomatic	74 (46.5)	65 (35.1)	
Comedo necrosis, *n* (%)			
No	60 (37.7)	34 (18.4)	< .0001
Yes	99 (62.3)	151 (81.6)	
Radiation, *n* (%)			
No	117 (73.6)	145 (78.4)	0.30
Yes	42 (26.4)	40 (21.6)	
Grade, *n* (%)			
1	25 (15.8)	0 (0.0)	< .0001
2	24 (15.2)	0 (0.0)	
3	109 (69.0)	185 (100.0)	
Margins, *n* (%)			
Negative	154 (97.5)	183 (98.9)	0.31
Positive	4 (2.5)	2 (1.1)	
Tumor size			
Median tumor size (range), cm	1.7 (0.1–14.5)	1.7 (0.2–12.0)	0.74
Size < 2.0, *n* (%)	88 (56.4)	101 (55.6)	
Size ≥ 2.5, *n* (%)	68 (43.6)	84 (45.4)	
Survival status, *n* (%)			
Alive	109 (68.6)	159 (86.0)	0.00
Dead	50 (31.4)	26 (14.0)	
10-year recurrence status, *n* (%)			
Recurrence free	128 (80.5)	159 (85.9)	0.18
Recurred	31 (19.5)	26 (14.1)	

Descriptive data detailing the training and validation cohort’s clinicopathological variables. The cutoff point for positive margins was 2 mm. In the training cohort, the tumor size of 3 cases was not known and a patient has missing data for margin status and grade. The proportional difference of clinicopathological variables are measured with the chi-square test

### Tumor slide selection

All diagnostic slides, from the lumpectomy surgical sample, for each patient were pathologist-reviewed (IMM and MST), and the best representative (to ensure the presence of adequate tumor tissue for analysis, morphological variation, and to confirm the pure DCIS diagnosis) formalin-fixed paraffin-embedded (FFPE) tumor blocks (donor) for each patient’s specimen were retrieved and included in the study. A fresh full-face section of 4 μm thickness was cut from each selected block, stained with H&E to standardize the consistency of staining quality, and again pathologist-reviewed (IMM and MST). Slide scanning was performed with a slide scanner using a × 40 magnification objective lens (0.24 μm/pixel) (Pannoramic 250 Flash III, 3DHISTECH) (Additional file 1: Supplementary methodology). Images were viewed at a maximum of × 400 magnification using a built-in functionality of image processing software (ImageScope, ver. 12.3.2.8013, Leica Microsystems). The slides were reviewed for image quality, those with out-of-focus areas re-scanned, and those with folded over tissues removed from the analysis.

### Automated full-slide annotation

OpenSlide software [43] allowed for 4× down-sampling of the full slides for computational feasibility. A simple graphical user interface (GUI) was developed to manually select and extract 50 × 50 pixel, pathologist-identified, “ground truth” image tiles from our training cohort, for training our annotation classifier to identify stroma, benign epithelial ducts (including normal breast parenchyma elements, epithelial hyperplasia, and other non-malignant epithelial changes), cancerous ducts, stromal regions with dense immune infiltration (immune cells occupying at least 50% of the tile area), and blood vessels (Additional file 2: Figure S1). The regions which fell outside these classes (such as areas of fat), or slide areas that were non-tissue, were given a background classification. An effort was made to select non-mixed-class (mutually exclusive) ground truth regions, which were completely surrounded by the pathologists’ manual annotation, with occasional edge cases (such as intersections of classes) being labeled by the predominant class in the image tile. Each 50 × 50 pixel image tile used was color normalized to a standard H&E staining distribution [44] to account for specimen and staining variability and to improve classifier performance [45]. The normalized image tiles were then color deconvoluted [46] into separate hematoxylin and eosin channels through an optical density matrix which contains the relative absorbance of each stain in the RGB color channel (Additional file 3: Table S1). A total of 166 texture features (Additional file 4: Table S2) were extracted from the deconvoluted hematoxylin (nuclear stain) channel for training the random forest annotation classifier. To reduce the same slide bias, testing of the classification ability was performed on a slide-based leave-one-out cross-validation. Each held-out set of image tiles used for testing was composed of (pathologist-annotated) ground truth regions from single individual slides, such that the test fold always consisted of extracted image tiles from a slide which was not used in training. The classifier was retrained with increasing tile *N* numbers in the training sets, until the cross-validated test set accuracy leveled off. To take into account the rotational invariance of the data (all of the image tiles have the same label regardless of the angle), and increase the size of the dataset, without decreasing the quality [47], we augmented the training image tiles by fourfold, by performing diagonal flipping, 90° rotation, and the combination of the two, on all training tiles. Tissue features extracted from the augmented set of image tiles were used to train a random forest classifier [48] for tissue annotation on the slide class (development depicted in Additional file 5: Figure S2A). The output of this random forest was the probability of the input image tile belonging to each of the five classes with the final assigned annotation determined by the highest probability.

Full slides being processed by the WSI pipeline (i.e., slides that were not previously used for training the annotation classifier) were annotated through a grid approach wherein adjacent non-overlapping 50 × 50 pixel image tiles (that made up the full slide) were processed (Fig. 1 (A/B/C)), as previously detailed for the training data, their features input into the trained random forest (Fig. 1 (D/E)), and the classified image tiles stitched together (Fig. 1 (F/G)). Additional post-processing, using neighborhood voting, was performed only for the analysis of spatial features (see the next section). In this approach, the class assigned to a region was amended if the sum of all its direct neighbors’ tree classifications resulted in a larger proportion vote for a different annotation (Additional file 6: Figure S3 shows an example).

### Full-slide feature optimization and recurrence prediction

Following the automated slide annotation, a set of distinct full-slide features can be extracted (Fig. 1 (I); Additional file 7: Table S3). The majority (99%) of these features consist of statistical moments (Additional file 8: Figure S4) of the 166 texture features for each annotated class and provide information on the shape of the texture feature distribution for that class. Additionally, spatial features were derived that related the distance and size of cancer to either the blood vessels or immune-rich stroma, as the literature suggests that both these spatial relationships have prognostic relevance (Additional file 9: Equation S1) [36, 49]. Finally, the proportions of each class, such as the amount of tumor on a slide (a quantity commonly calculated in cancer staging), and average annotation confidence (calculated by averaging the number of trees which voted for each annotated class, such that low values would be given if there was a large ambiguity for any annotation on that slide) were included as features. To reduce data dimensionality and improve training time and prediction accuracy [50], a feature reduction step was performed. First, we selected a maximum follow-up time point past which a patient will be right censored and considered as a non-recurring patient (Additional file 1: Supplementary methodology). For the selected follow-up time, we filtered and sequentially selected the list of candidate features within multiple machine learning models, trained with uniform (equal) prior class probabilities, and used patient recurrence status as the input label, to build an optimized classifier (Fig. 1 (J); Additional file 1: Supplementary methodology). The performance of this final DCIS recurrence risk classifier model was then examined univariately through Kaplan-Meier curves (Fig. 1 (K/L)). This model outputs a prognostic risk on a slide level. For the patients with multiple slides (*n* = 127 in this cohort), if any of their slides were classified as high risk, those patients were given a high-risk classification (Additional file 10: Figure S5). For comparison, we performed a separate analysis wherein we omitted these patients to test if the model performance suffered. The development of this full slide classifier is depicted in Additional file 5: Figure S2B.

To test the feasibility of a continuous metric, we separately (a) used the trained random forest class probability output (which signifies the proportion of trees voting for a class, e.g., recurrence), rather than the corresponding binary (high versus low risk, normally split by the majority vote of the aforementioned proportion) classification, and (b) trained a random survival forest (RSF) [51, 52] that provided each patient a “risk score” which was equal to 1—the RSF’s output survival function for that patient.

### Comparison of recurrence classifier accuracy with or without inclusion of standard clinicopathologic variables

To evaluate if our final model provides an advantage over DCIS recurrence risk prediction using available clinicopathologic parameters (comedo necrosis, size, grade, surgical margins, and patients age), we (a) performed multivariable Cox proportional hazard regression analysis using these clinicopathologic variables as covariates and (b) concatenated the clinicopathologic variables to the 8 (optimized) features in our model and assessed the performance of this expanded machine learning model, and the importance of each variable to the overall prediction accuracy of this model, via a variable permutation approach.

### Prediction of DCIS recurrence risk in the context of different adjuvant therapies

We then evaluated our final model’s ability to predict DCIS recurrence risk among patients who (a) were diagnosed as having high-grade DCIS (due to the clinical relevance), (b) were treated with BCS alone, and (c) received adjuvant radiotherapy after BCS. The risk of invasive recurrence was also analyzed within the classified patient risk groups.

### Recurrence classifier validation

To validate the recurrence classifier’s significant prognostic ability, we applied it to a second independent cohort of BCS-treated patients diagnosed with high-grade pure DCIS. The final feature-selected recurrence risk classifier model and pipeline, as previously trained for both annotation and recurrence classification, was used on 199 slides (of 185 patients, which were not included in the training cohort). The patients predicted by the model to be in the high-risk subgroup were compared with patients predicted to be in the low recurrence risk subgroup through survival analysis (Kaplan-Meier and Cox regression) of their 10-year recurrence outcomes (Additional file 5: Figure S2C).

### Statistical analysis

Statistical analysis was carried out with SAS 9.4 software (Cary, NC, USA), MATLAB R2017b (Natick, MA, USA), the Python programming language (Python Software Foundation, https://www.python.org/), and R (R Foundation for Statistical Computing, Vienna, Austria, http://www.R-project.org/). The significance of the texture feature differences between annotated classes was analyzed with an analysis of variance (ANOVA) with a post-hoc Tukey-Kramer procedure. Two-tailed *t* tests were used during the initial stage of feature selection and for comparing the significance of the continuous metric values. The accuracy metric was calculated as the sum of true positives (TP) and true negatives (TN) divided by the total observations. The “positive” class in the recurrence analysis comprised patients who experienced recurrence within 10 years of diagnosis, and the “negative” class was composed of patients who were censored. True-positive (TP) patients were those in the high-risk group who indeed experienced recurrence. True-negative (TN) cases were those in the low-risk group who were censored. False-positive (FP) patients were recurrence-free patients in the high-risk group, and false-negative (FN) patients were patients classified as low risk who recurred. Additional confusion matrix performance metrics used were sensitivity (Sn: TP/(TP + FN)), specificity (Sp: TN/(TN + FP), positive predictive value (PPV: TP/(TP + FP)), negative predictive Value (NPV: TN/(TN + FN)), and odds ratio (OR: (TP/TN)/(FN/TN)). The accuracy for the training recurrence classifiers was ascertained through the average of 100 repeated fivefold cross-validation, with confusion matrices chosen from the combined testing folds of one of the repeats. When analyzing the invasive or DCIS recurrence separately, patients who experienced DCIS or invasive recurrence were treated as censored. For the training cohort, both the Kaplan-Meier survival analysis and the subsequent multivariate analyses were performed on the fivefold cross-validated data with risk classification groups taken from the cross-validated test sets [53] and significance determined using the log-rank test and Wald chi-square test, respectively. Mean recurrence-free survival estimates were calculated by taking the area under the survival curves [54]. Comparisons between the clinicopathological proportions of training/testing and the validation cohort were carried out through a chi-square test. Multivariate analysis was controlled for comedo necrosis, size, grade, age, and the surgical margin status. Model fit was compared through the Akaike Information Criterion (AIC) [55], a measure of goodness of fit/efficiency within the Cox regression statistical model. The lower the AIC value, the better the likelihood. Model discrimination ability was analyzed through the Harrell’s *c*-statistic [56] using a SAS macro [57]. Feature importance within the RF model that included standard clinicopathologic variables concatenated with the features in our recurrence classifier was determined through 100 iterations of the out-of-bag variable permutations in which the average increase in prediction error, for each variable whose value was permuted, was calculated for the out-of-bag observations [48]. For fitting and optimizing the survival forest model, the R package “randomForestSRC” [58] was used. When necessary, dichotomization of continuous features was performed by identifying an optimal outcome-based threshold [59]. To facilitate visualization of hazard ratios for continuous variables, *z*-score transformation of features was used.

## Results

### Traditional clinicopathological factors have limited DCIS recurrence risk predictive ability

The major clinicopathological characteristics for the cohorts of DCIS patients used to train and validate our model are shown in Table 1. For the training cohort, while the recurrence rate was low (23%), the majority (84%) of recurrences occurred within the first 10 years of follow-up (Additional file 11: Figure S6). Patients were mostly high-grade (69%), post-menopausal (80.5%), older than 50 (83.7%), and did not receive radiotherapy (73.6%). Additionally, almost all patients had a complete excision with wide (> 2 mm) negative margins (97.5%). Within this training cohort, aside from an increased prevalence of high grade, patients who developed recurrence did not have any significant differences in the proportions of standard clinicopathological variables compared to patients who remained recurrence-free (Additional file 12: Table S4). The validation cohort consisted of only high-grade (3) patients, but otherwise differed from the training cohort with higher rates of comedo necrosis (81.6%, *p* < 0.0001), and a slightly higher proportion of patients presenting at screening (64.9%, *p* = 0.0316) (Table 1). Within this validation cohort, only radiation has a significant proportional difference between patients who developed recurrence and those who did not (Additional file 13: Table S5).

### Texture features differentiate significantly between annotated tissue regions

**Fig. 2:**
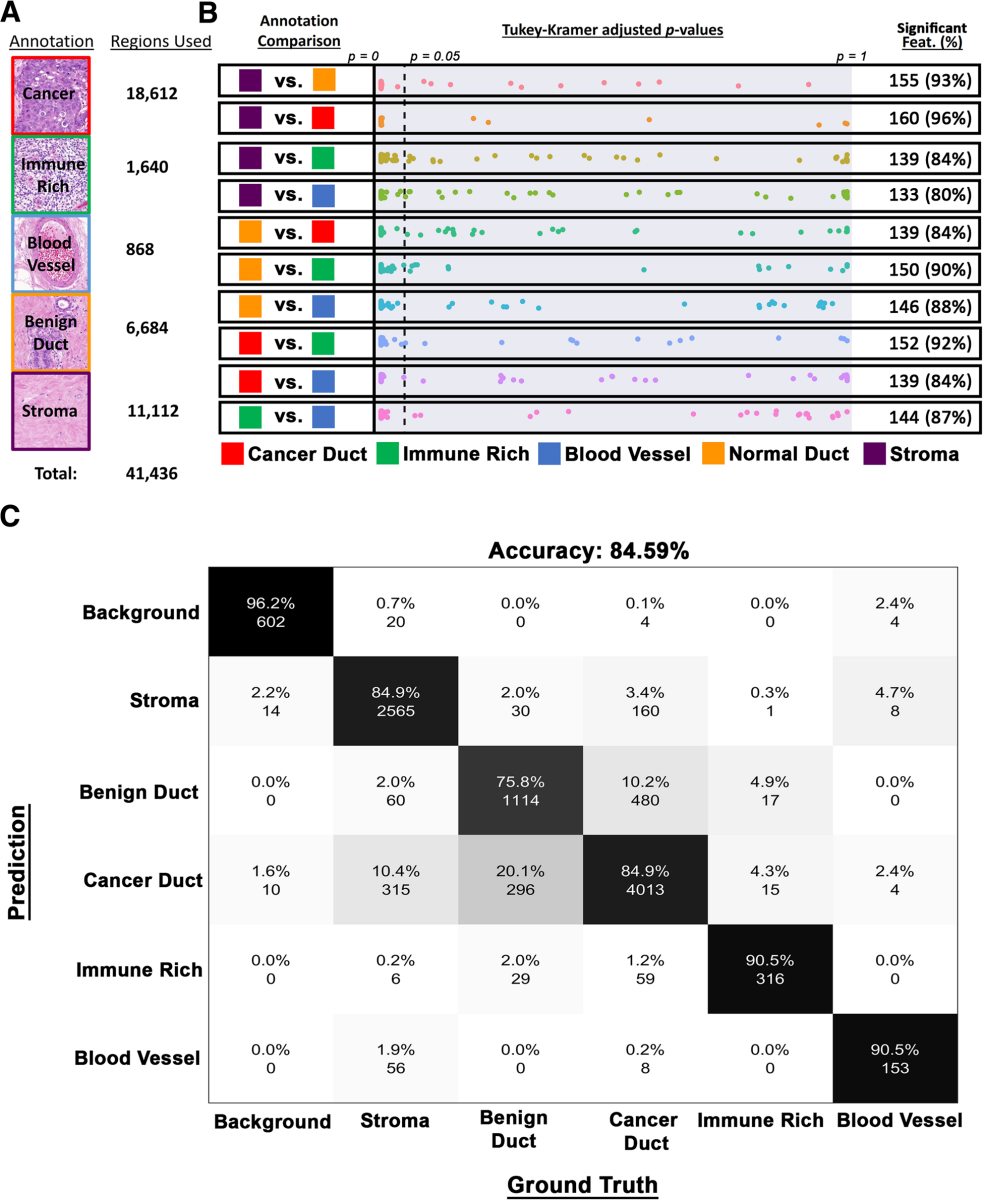
Full-slide annotation. **a** List of annotation classes used, and representative examples, alongside the number of ground truth regions available to develop the texture-based annotation classifier. **b** Multivariate-adjusted *p* value (Tukey-Kramer) distributions for all 166 features (as points) between all annotated class comparisons. Reference dotted line indicates an adjusted *p* value of 0.05, with features possessing the significant discriminatory ability (*p* values < 0.05) situated on the left of it and summarized alongside. **c** Confusion matrix (which quantifies the performance of the class annotation model) comparing the training ground truth data to the cross-validated annotation classifier test set outputs. The analysis was performed on the original regions before fourfold augmentation

To develop a pipeline for automated annotation of various clinically relevant regions within DCIS tumor tissue sections, we found that the overall accuracy leveled off at 10,359 50 × 50 pixel ground truth image tiles (Additional file 14: Figure S7) from 32 training cohort slides. For developing the final annotation classifier, these ground truth areas were augmented (using rotation/transposition) to a total of 41,436 (Fig. 2a). Using the original (non-augmented) collection of ground truth regions, we observed that the majority of our texture features possessed significant discriminatory ability between all annotated class combinations (Fig. 2b). The classes with the most discriminatory texture features between them were cancer versus stroma (96% of features had a *p* value < 0.05). By contrast, texture features had the least discriminating power when it came to distinguishing stroma from the blood vessels (only 80% of features were significant). Cross-validation of the unaugmented ground truth collection resulted in an accuracy of 84.59%, with individual class distinction accuracies, not counting background, ranging from 75.8 to 90.5% (Fig. 2c) (with additional performance metrics shown in Additional file 15: Table S6).

### An eight-feature recurrence classifier significantly predicts recurrence risk

Thresholding at a 10-year follow-up maximized the number of significant whole-slide features different between slides from patients who recurred versus those that did not progress (Additional file 16: Figure S8A). This follow-up time is also consistent with many follow-up times in clinical studies [60] and with the fact that most DCIS patients recur within 10 years. Overall, around 1238 (37%) whole-slide features differed significantly (*p* < 0.05) with a 10-year follow-up as compared to at most 25% for 5-, 15-, and 20-year follow-up time points.

**Fig. 3:**
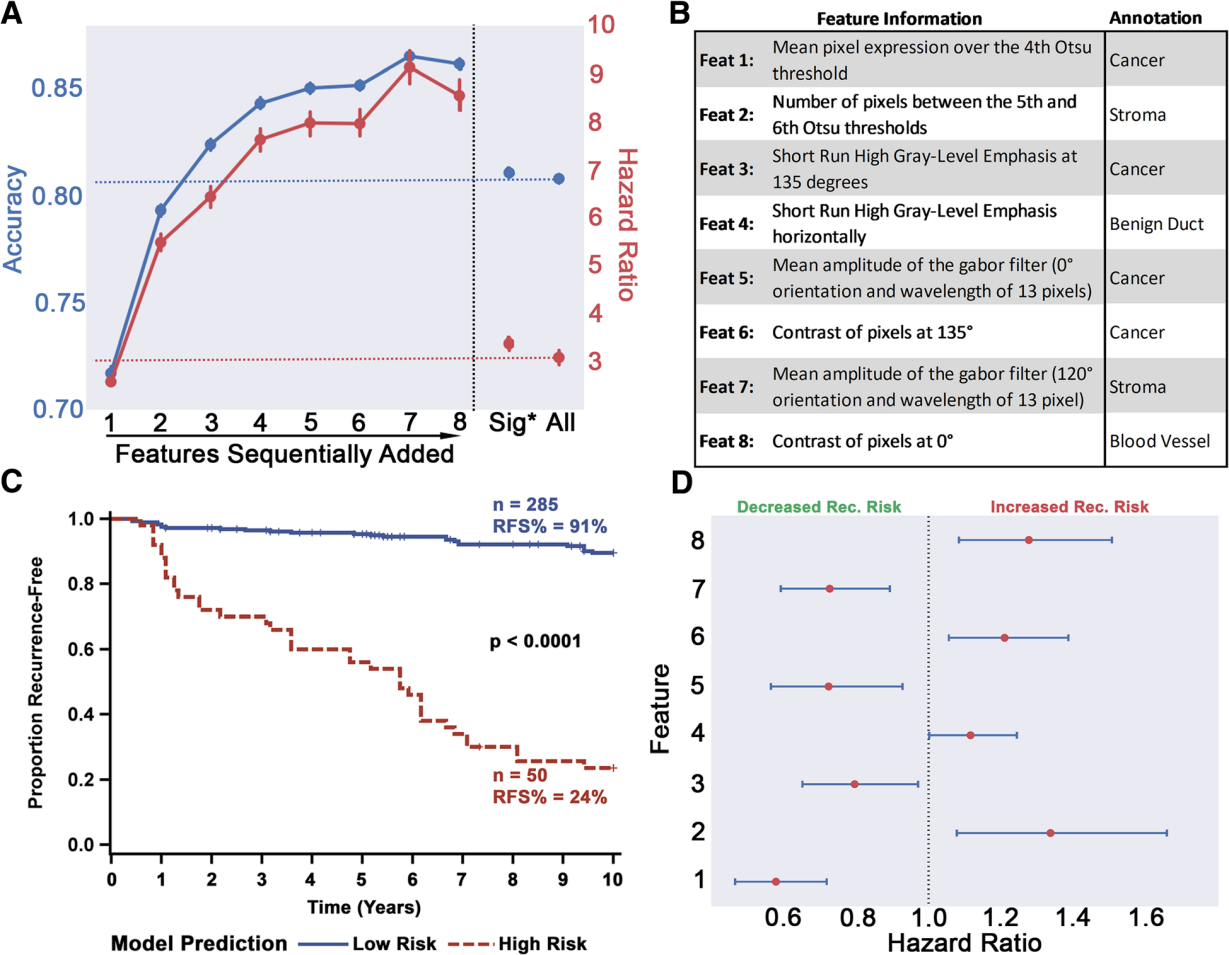
Full-slide feature selection for the development of recurrence classifier. **a** The change in model accuracy and high-risk group hazard ratio with the sequential addition of features. The reference hazard ratio and accuracies, based on the model with all features, are shown in red and blue horizontal dashed lines, respectively. The model which included all filtered features (Sig*: *p* < 0.05) is also shown for comparison. Bars on markers indicate 95% confidence intervals. **b** General feature descriptions, and the annotations from which they stem from, of the final 8-feature recurrence classification model. **c** Kaplan-Meier curves showing stratification of patient slides by the final recurrence classifier model. Data shown is based on the slides used for the training cohort, wherein the test sets for each selected cross-validated iteration were combined. Significance was measured using the log-rank test. **d** Univariate HR of the selected features, *z*-score transformed for illustrative purposes. All variables are significant, and blue horizontal lines depict 95% confidence intervals. The fact that none of the confidence intervals cross the HR = 1.0 reference line shows that these features are highly and unequivocally significant

Testing 10-year recurrence risk model built with these filtered features (i.e., using all significant features prior to the sequential removal step in Fig. 1 (J)) resulted in an average fivefold cross-validated accuracy around 80%, regardless of the ML model (Additional file 17: Table S7) and a random forest high-risk group possessing a hazard ratio of 3.19 (Fig. 3a), almost equivalent to the performance of using the full feature set (accuracy 80.8%; HR 3.13). Interestingly, among the filtered whole-slide features, the majority (88%) stemmed from non-cancer annotations and only 1% came from the differences in lymphocyte-dense properties between patients (Additional file 16: Figure S8B). Choosing the most prognostic variables through the sequential forward selection, though, resulted in half of the features being derived from cancer areas (Fig. 3b with additional feature details in Additional file 18: Table S8). The final 8-feature model lowered the misclassification rate to 0.101, achieved an average (of 100 iterations) cross-validated accuracy above 86%, and yielded a model that robustly stratified the DCIS patients in our training cohort and identified a high-risk group with 8.5× higher recurrence risk by 10 years (Fig. 3a). Figure 3c illustrates a typical Kaplan-Meier survival curve from one of the model training iterations (out of the total 100) of the combined cross-validated test sets. The slides classified into the high-risk group carry a recurrence-free survival (RFS) of only 24% compared to the 90% seen in the low-risk group. To show the importance of the initial machine learning annotation step (Fig. 1 (A–G)), a “non-annotated” RF model built (with feature selection) without utilizing the annotation classification (simply using the overall texture statistical moments of all the areas of the slides) resulted in a significantly lower accuracy (79%) and HR (2.82) (Additional file 17: Table S7).

**Fig. 4:**
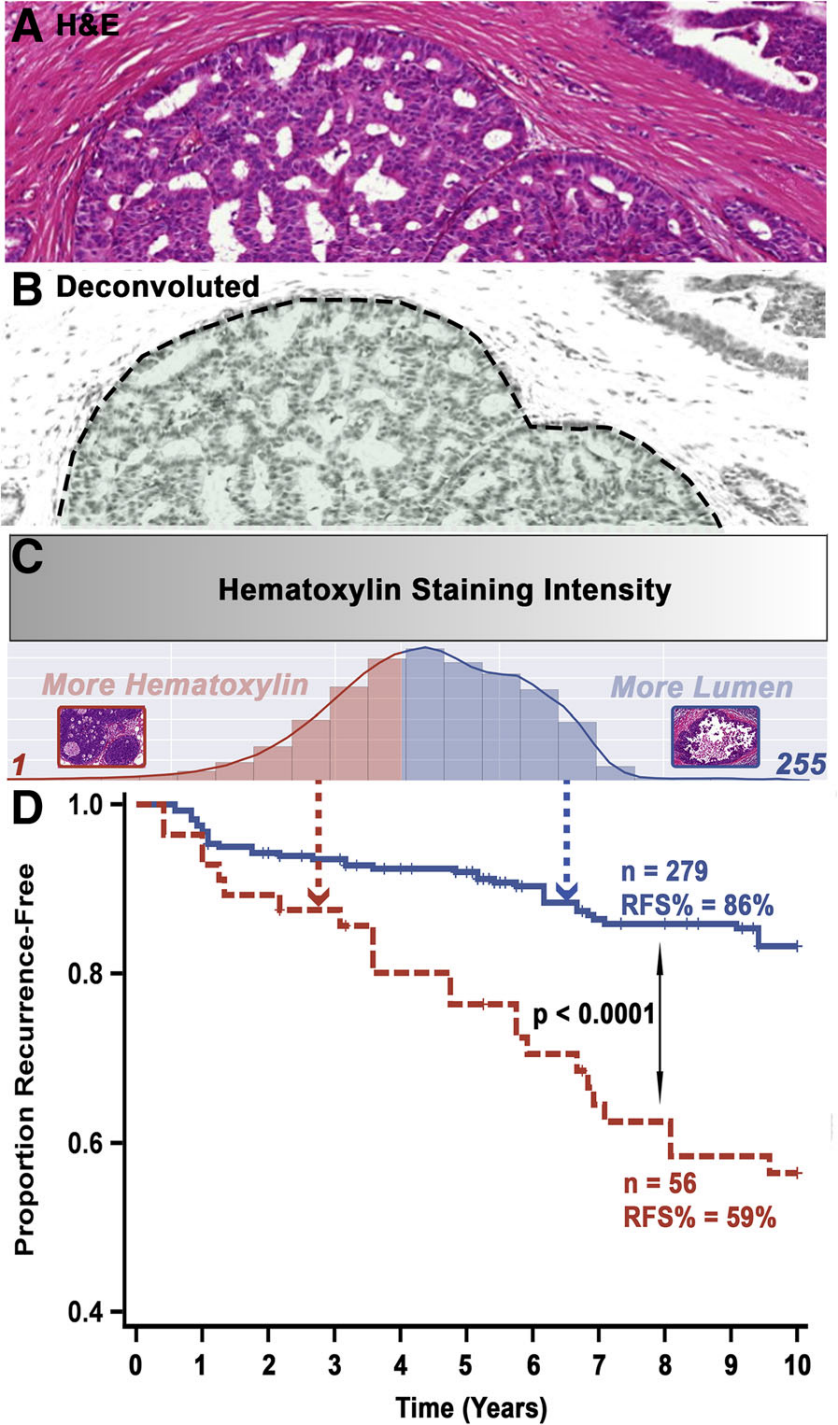
Interpretation and prognostic relevance of the most prognostic feature in our eight-feature DCIS recurrence risk prediction model. **a** An example “cancer” region with a cribriform architecture in an H&E-stained slide (prior to deconvolution). **b** The region shown in **a** after hematoxylin deconvolution. **c** Intense hematoxylin staining (relative to the image tile section) is represented by a gray-level intensity of 1, while no staining is depicted by a gray-level value of 255. The adaptive Otsu thresholds by progressively using a higher threshold. Therefore, if the cancer region has lumens, it would yield a higher average intensity (more white pixels) as compared to a solid pattern (no white pixels). Using an optimized threshold of 208, it is observed that full slides whose cancer regions have an average feature #1 above that cutoff recur significantly less than patients below that threshold (**d**)

The eight features selected for the final model, when evaluated as continuous variables in univariate analysis, all provided significant prognostic value, with half being associated with a higher risk of recurrence and the other half providing a protective effect (Fig. 3d). Dichotomizing patients into groups using the two mean cancer features (consisting of feature #1 and #3, as the mean moment and cancer annotations are the most intelligible combination for texture-based analyses), for interpretive purposes, showed conflicting effects. Alone, feature #1, calculates the hematoxylin staining, or blue color intensity, per pixel (or point) within the malignant ductal profile areas (above a certain Otsu method autogenerated threshold [61]) (Fig. 4a–d), very significantly stratified patients into two distinct risk groups (Fig. 4d), while feature #3 was unable to do so (Additional file 19: Figure S9A). However, if patients were first split into high- and low-risk groups through feature #1 (Additional file 19: Figure S9B) followed by another stratification using feature #3, a significant difference in survival between the two subgroups was increased when compared to the stratification by feature #1 alone (Additional file 19: Figure S9C), showing the dependency of variables for maximizing prognostic relevance (high-risk group HR for feature #1 alone = 3.017, high-risk group HR for features #1 + #3 = 7.308).

**Fig. 5:**
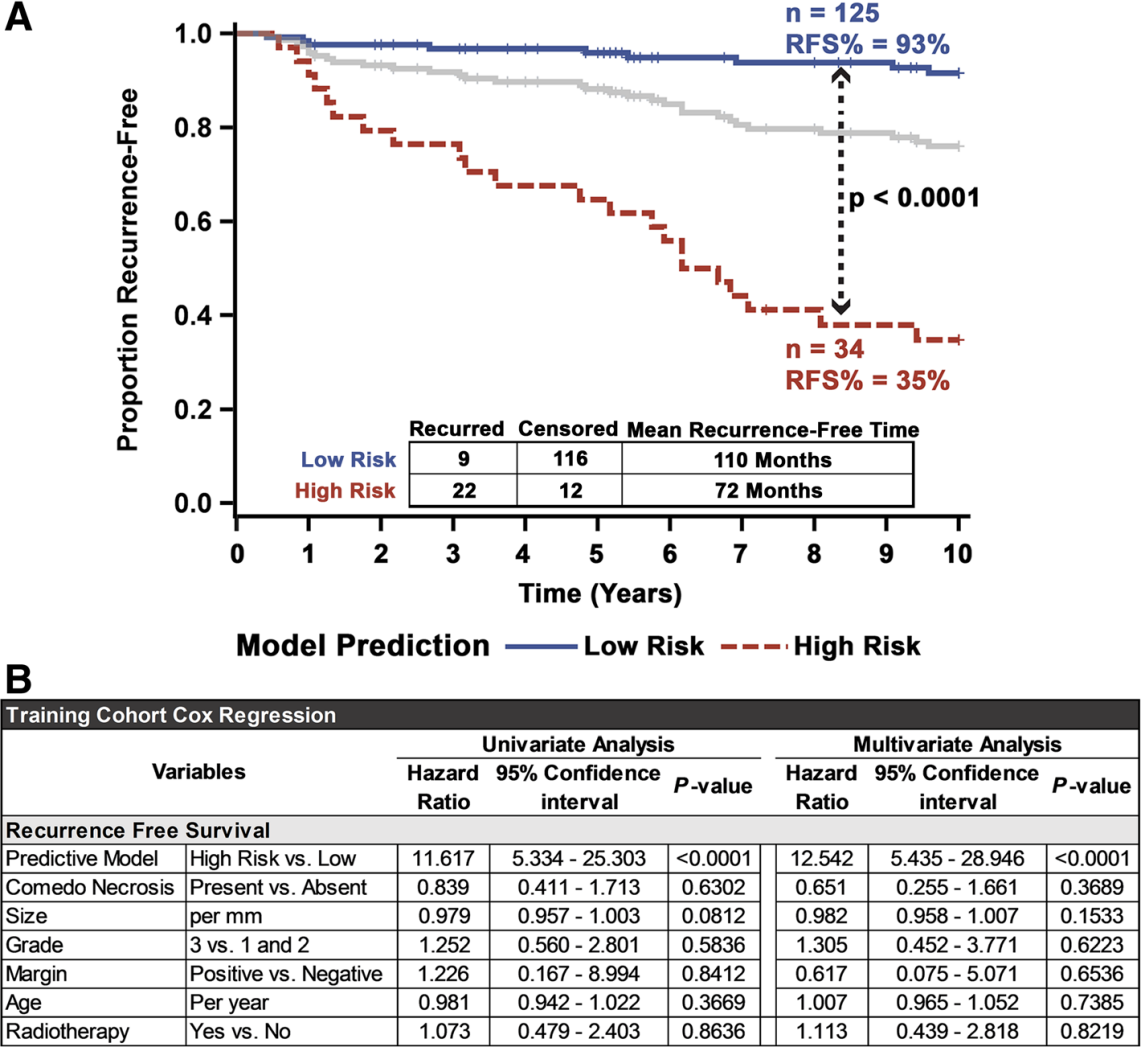
Univariate and multivariate analysis of the eight-feature DCIS recurrence risk prediction model on the training cohort. **a** Fivefold cross-validated Kaplan-Meier curves of the training cohort. Significance is measured using the log-rank test, and the gray line represents the unstratified full cohort. **b** Univariate and multivariate Cox regression analysis comparing the influence of common clinicopathological variables alongside the eight-feature recurrence risk prediction model for recurrence-free survival, on the training set (after fivefold cross-validation)

Applying the recurrence classifier based on the final eight features at the patient level showed that the classifier significantly stratified the patients in the training cohort (*p* < 0.0001). Patients classified to the high-risk group (*N* = 34) had an RFS of only 35% (with a mean recurrence-free time of 72 months), compared to the 93% (mean recurrence-free time of 110 months) seen in patients in the low-risk group (*N* = 125) (Fig. 5a). This significant stratification remained even if the analysis was performed after omitting patients with discordant slide classifications (Additional file 20: Figure S10). This iteration had a univariate high-risk hazard ratio of 11.6 and retained its very high significance when controlling for necrosis, size, grade, margins, radiation therapy, and patient age (Fig. 5b). None of the clinical variables in the original cohort showed significant risk stratification ability in multivariate analysis, although grade was significant univariately (Fig. 5b and Additional file 21: Figure S11). Moreover, the model provided a superior *c*-index (0.77), model fit (AIC = 239.8) (Additional file 22: Figure S12), accuracy (0.87), specificity (0.91), PPV (0.65), NPV (0.93), and OR (23.6) (Table 2) to the clinical variables. However, the model produced a lower sensitivity (0.71) compared to grade (0.74) and age (0.77). Additionally, select clinical variables neither improved the overall model nor add any prognostic relevance individually (Additional file 23: Figure S13). Notably, the same model was able to significantly stratify high-grade DCIS patients (Additional file 24: Figure S14A), low/intermediate-grade DCIS patients (Additional file 24: Figure S14B), the subset of all patients who received adjuvant radiation therapy, and all patients treated with BCS alone (Additional file 24: Figure S14C and D) into the subgroups with high and low recurrence risks. Additionally, the model was able to identify patients at high-risk for both invasive (Additional file 25: Figure S15) and DCIS recurrence (Additional file 26: Figure S16), even when controlling for clinicopathological variables. Transforming the binary classification of the model to a continuous measure, equaling the proportion (multiplied by 100) of trees which voted for the “recurrence” class, resulted in a significantly higher (*p* < 0.0001) average score for slides which came from patients who recurred within 10 years (45.8) versus those who did not (21.6) (Additional file 27: Figure S17A). Similarly, producing a continuous metric, through training an RSF using the selected eight features, produced an average score of a slide from a patient who eventually recurred (34.3) that was significantly higher (*p* < 0.0001) than those who did not (19.3) (Additional file 27: Figure S17B). Additionally, both continuous models provided prognostic significance (*p* < 0.0001), with a unit increase of class probability providing incremental 5.6% higher 10-year recurrence risk and a 5.1% increase through the RSF (Additional file 28: Table S9).

**Table 2:** Model performance

Model and Clinicopathologic Variables 2x2 Performance Metrics										
	Training Cohort					Validation Cohort				
Variable	Rec. Status at 10 Years			Metrics		Rec. Status at 10 Years			Metrics	
Model		Censored	Recurred	Acc: 0.87	PPV: 0.65		Censored	Recurred	Acc: 0.85	PPV: 0.46
	Low Risk	**116**	9	Sn: 0.71	NPV: 0.93	Low Risk	**144**	13	Sn: 0.50	NPV: 0.92
	High Risk	12	**22**	Sp: 0.91	OR: 23.6	High Risk	15	**13**	Sp: 0.91	OR: 9.60
Necrosis		Censored	Recurred	Acc: 0.41	PPV: 0.18		Censored	Recurred	Acc: 0.26	PPV: 0.13
	No	**47**	13	Sn: 0.58	NPV: 0.78	No	**28**	6	Sn: 0.77	NPV: 0.82
	Yes	81	**18**	Sp: 0.37	OR: 0.80	Yes	131	**20**	Sp: 0.18	OR: 0.71
Size		Censored	Recurred	Acc: 0.50	PPV: 0.15		Censored	Recurred	Acc: 0.49	PPV: 0.10
	Below	**68**	20	Sn: 0.33	NPV: 0.77	Below	**83**	18	Sn: 0.31	NPV: 0.82
	Above	58	**10**	Sp: 0.54	OR: 0.59	Above	76	**8**	Sp: 0.52	OR: 0.49
Age		Censored	Recurred	Acc: 0.27	PPV: 0.18		Censored	Recurred	Acc: 0.24	PPV: 0.15
	Below	**19**	7	Sn: 0.77	NPV: 0.73	Below	**21**	2	Sn: 0.92	NPV: 0.91
	Above	109	**24**	Sp: 0.15	OR: 0.60	Above	138	**24**	Sp: 0.13	OR: 1.83
Radiotherapy		Censored	Recurred	Acc: 0.64	PPV: 0.19		Censored	Recurred	Acc: 0.66	PPV: 0.05
	No	**94**	23	Sn: 0.26	NPV: 0.80	No	**121**	24	Sn: 0.08	NPV: 0.83
	Yes	34	**8**	Sp: 0.73	OR: 0.96	Yes	38	**2**	Sp: 0.76	OR: 0.27
Grade		Censored	Recurred	Acc: 0.41	PPV: 0.21	*				
	I/II	**41**	8	Sn: 0.74	NPV: 0.84					
	III	86	**23**	Sp: 0.32	OR: 1.37					

The 2 × 2 confusion matrix and performance metrics for the 8-feature model and common clinopathological variables in the training and validation cohorts. For each variable, the positive condition was recurrence within 10 years. A 2 × 2 matrix for grade in the validation cohort was omitted due to all patients belonging to grade III. Margin status was not shown for either cohort due to almost all patients having negative margins. The threshold used for patient age was 50 years, and the threshold for size was 2 cm

### Validation study confirms prognostic value of the eight-feature recurrence risk classifier

**Fig. 6:**
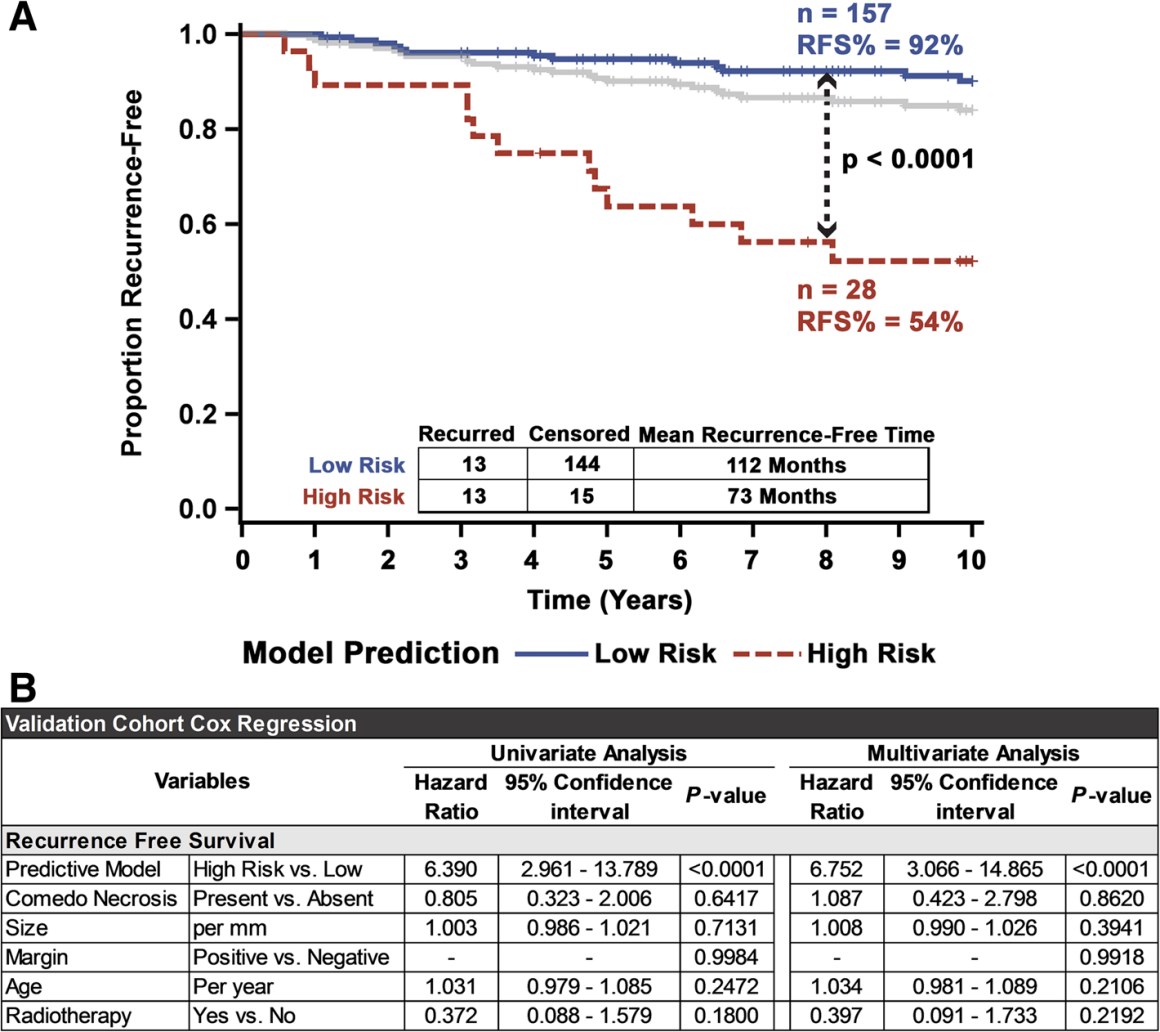
Validation of 8-feature DCIS recurrence risk prediction model in an independent validation cohort. **a** Kaplan-Meier curves showing a robust stratification of patients in the validation cohort into high risk of recurrence and low risk of recurrence subgroups. Significance was measured using the log-rank test, and the gray line represents the unstratified full validation cohort. **b** Univariate and multivariate Cox regression analysis of the validation cohort comparing the influence of common clinicopathological variables on the recurrence risk predictive 8-feature model, for 10-year recurrence-free survival

We proceeded to validate our eight-feature DCIS recurrence risk prediction model in an independent validation cohort of DCIS cases (*n* = 185 from Nottingham University Hospital). Analyzing individual slides (treating each slide as an individual patient) using our previously trained eight-feature classifier resulted in highly significant stratification of the validation cohort into high- and low-risk groups with regard to their RFS (Additional file 29: Figure S18). A patient-wise analysis led to further improvement in recurrence risk prediction. Ninety-two percent of patients classified into the low risk stayed recurrence-free for 10 years (mean recurrence-free time of 112 months), compared to only 54% (mean recurrence-free time of 73 months) for patients who are classified as high risk (Fig. 6a). Removing patients with discordant cases did not adjust the model stratification (Additional file 30: Figure S19). While lower than the training/test cohort, the univariate hazard ratio of this classifier on the validation cohort patients is 6.4 (*p* < 0.0001) and over 6.8 (*p* < 0.0001) when controlling for necrosis, size, margin status, and age (Fig. 6b). Once again, the model provided superior concordance (*c*-index = 0.69), model fit (AIC = 243) (Additional file 31: Figure S20), and most traditional 2 × 2 performance metrics (Acc = 0.85, Sp = 0.91, PPV = 0.46, NPV = 0.92, OR = 9.6) (Table 2), as compared to the clinicopathological variables, but had lower sensitivity (0.5) compared to age (0.92) and necrosis (0.77). Even though this validation cohort had very few patients recurring after radiotherapy, the eight-feature recurrence risk predictive model was able to significantly predict long-term outcomes after radiotherapy (Additional file 32: Figure S21A). Additionally, a clear high-risk subgroup was identified among patients treated with only BCS (Additional file 32: Figure S21B). Censoring the eight patients whose recurrence was DCIS (rather than invasive disease) resulted in a robust identification of patients at high risk of recurrence as invasive disease, regardless of other clinicopathological variables (Additional file 33: Figure S22). Furthermore, although the number of events was limited, the model significantly identified a group at high risk of DCIS recurrence (Additional file 34: Figure S23). Using this model’s continuous class probability showed a significantly higher proportion of recurrence voting trees (45.8) for patients who eventually had 10-year recurrence versus those that did not (26.8) (Additional file 5: Figure S2A). This score was significantly prognostically (*p* < 0.0001), providing 3.6% incrementally increase risk for 10-year recurrence (Additional file 28: Table S9).

Equivalently, using an RFS model for continuous risk also resulted in a significant (*p* = 0.0358) hazard ratio (HR = 1.05 per unit increase) (Additional file 28: Table S9). However, while the mean slide score (44.6) for a recurred patient slide was statistically significantly higher (*p* = 0.0355) than a slide from a patient who does not recur (42.0), this difference was much smaller than the difference in scores observed between the recurred and non-recurred slides in the training cohorts (Additional file 5: Figure S27B). Furthermore, the average scores of the RFS model on the validation cohort were both much higher than the average scores in the training cohort (*p* < 0.05) (Additional file 5: Figure S2B).

## Discussion

Limited understanding of the progression of pre-invasive ductal lesions to invasive ones and lack of clinicopathological [62] and molecular markers [12], which can predict recurrence, lead to uncertainty in therapeutic decision-making. Without a confident measure of recurrence risk, patients are often at risk for over- and under-treatment [41]. The aim of this study was to develop a novel image analysis pipeline which could predict the 10-year ipsilateral recurrence risk in DCIS patients treated with BCS. We also show that our approach of class-annotating slide regions prior to feature extraction for recurrence prediction enhances our model’s performance. While the increase in the accuracy from using an annotation step was only moderate, the hazard ratio, and therefore prognostic value, increase was substantial. Additionally, this initial annotation classification enables better interpretation of the features that our model uses for recurrence prediction; this is particularly important given that with machine learning approaches, it is often difficult to understand why the trained model responds in a particular way to a set of input data. It would be interesting to test whether adding more classes leads to an improved performance of the model.

Predictably, most of the features selected for the final recurrence classifier model originate from tumor regions, whose cells show both gross morphological changes and nuclear alterations, such as deviations in heterochromatin [63]. The patterns and distribution of hematoxylin within cancer could reflect changes in both ductal architecture and cellular cytological features, both long mainstays of DCIS grading [64–71], and can be continuously quantified [30]. The surrounding stroma is composed of a collection of many varied cell types that also produce diverse hematoxylin staining patterns. Fibroblasts [34] and myofibroblasts [72], for example, have both been implicated in DCIS invasion and recurrence and provide distinct hematoxylin distributions. As fibroblasts are rich in rough endoplasmic reticulum, they would be much more basophilic [40] and demonstrate different hematoxylin staining patterns compared to myofibroblasts. It should be noted, as a limitation, that the stroma is the principal area where the addition of eosin deconvolution into our pipeline would perhaps improve model performance due to stromal collagen diffusion and densities. Thickening of the ECM, through fibrous deposits such as collagen, promotes cancer progression [73], and since collagen is eosinophilic, its distribution and texture features would be best quantified with the eosin stain.

Benign epithelial ducts and blood vessels both provide a single feature towards the final recurrence classifier model. These classes’ relative deficiency of selected features can perhaps be due to the limitations for this annotation within the pipeline and/or these regions not being as prognostically informative as compared to cancer or the surrounding stroma. Vascular heterogeneity has a varied impact on breast tumor progression [74]. It is possible that this prognostic value is being harnessed through our recurrence classifier. However, our choice of H&E slides limits us to only studying the texture of the vessels containing visible red blood cells within a relatively large section (image tile); a smaller sliding window would perhaps uncover smaller, but relevant, vascularization. It is interesting that a feature of benign epithelial ducts was included in our final recurrence classifier. As our use of the “benign epithelial duct” annotation is inclusive of everything but DCIS, it is possible that potentially prognostic information inherent in regions containing abnormal malignancy precursor cells is being captured by our feature. Proliferative, non-cancerous alterations such as columnar cell lesions often co-occur with DCIS, suggesting their potential for malignant transformations and can be used as a marker for BC risk [75]. Importantly, these premalignant regions could also possess variation in hematoxylin staining patterns. For example, usual ductal hyperplasia [37] characteristically shows nuclear pseudo-inclusions [76], which would show a unique hematoxylin texture pattern. As the distinction between some benign areas and low-grade DCIS is not clear [77], with potentially similar histological and nuclear features, it comes as no surprise that benign epithelial ducts and cancer duct annotations had a level of uncertainty. Further testing to differentiate annotations between non-benign and benign regions might be advisable to see if this distinction can glean additional prognostic and interpretable value. Immune-rich regions were notably absent in both filtered features and the final model, likely due to the immune dense areas of lymphocyte infiltration not possessing significant variability in cell and nuclear morphology [78].

Based on the hematoxylin texture distribution of these annotated regions, our model consists of some features that are perhaps amenable to logical interpretation in terms of disease biology, and some that elude obvious explanation; yet, both types are useful prognostically. Interpretable texture features can correlate with accepted pathological principles, such as histology, and allow for a continuous, quantifiable, and non-biased measure which is beyond the capacity of the human eye. Additionally, they instill more confidence in machine learning approaches, which often can be considered as black boxes. On the other hand, texture features and patterns which may lack discriminatory ability per se can still provide discriminatory information when their higher order spatial statistics (e.g., statistical moments) are considered [79]. These non-visually extractable features can supplement a pathologist’s visual inspection to provide additional unbiased prognostic value [80]. Our final full-slide recurrence classifier model includes both types of features, with a clear example demonstrated through the two mean cancer slide-annotated textures (the more interpretable feature #1, and a less intuitively interpretable feature #3). The most significant feature in the model (i.e., feature #1) quantifies the average hematoxylin intensity at a high-end threshold, which broadly represents the underlying average tissue architecture (by enabling luminal versus more solid areas to be distinguished), long shown to have some value predicting DCIS recurrence [81]. Furthermore, as this feature is a continuous measurement, it also presents a relative scale that a more broadly defined architectural pattern (such as a classification of cribriform architecture) cannot. This can be especially useful for comparing between mixed pattern cases, which are often present in DCIS [82] and underlie inter-observer variability among pathologists [83]. Our univariate analysis indicated that a lower value of feature #1 correlated strongly with a higher rate of recurrence, consistent with the empirical observation that more solid DCIS cases have poorer outcomes [81] and are often of higher grade [82]. Feature #3 on the other hand does not grant such discernable interpretation for our data. The short runs high gray-level emphasis (SRHGE) is a second-order texture feature that explains the joint distribution of spatial arrangement and gray level, which, notwithstanding, has had previous success in machine learning algorithms for cancer classification [84–86]. Interestingly, this feature also presents a prime example of the dependency of some of these features within our data and why a tree-based classifier can exploit such a relationship. On its own, feature #3 did not show significant stratification ability; however, if used on patients directly after splitting them into high and low feature #1 groups, we observed a marked increase in stratification ability. This type of association is conserved in a tree-based algorithm as they allow for branching results which depend on upstream features.

In this study, we used a combination of eight features to create a machine learning-based model to predict the risk of DCIS recurrence. Our model demonstrated outstanding prognostic ability in two independent patient cohorts, commandingly outperforming traditional histopathological variables in most traditional performance metrics (accuracy, specificity, PPV, NPV, and OR). While some variables had superior sensitivity (age and grade in the training cohort and necrosis and age in the validation cohort) to the recurrence model, and improving these metrics represents an ongoing challenge of the model, these variables also had much lower PPV, suggesting that being in high risk in the model still provides much higher discriminatory ability with identifying patients who develop recurrence. Additionally, this model was able to create prognostic groups with over double the hazard ratio of risk groups created through the commercially available Oncotype DCIS score [87] and improved concordance to the DCIS nomogram [24]. In our validation cohort, the model was able to identify a high-risk group of patients that had almost a 50% chance of recurring within 10 years (versus < 10% chance within the low-risk group).

Within the subsets of patients treated with BCS alone or those receiving additional adjuvant radiation, the recurrence classifier model also identified patients likely to recur. Thus, our model can serve as a clinical tool to help with treatment decisions. For example, high-risk patients who may have undergone BCS alone might require more aggressive treatments (such as radiotherapy) to avert the recurrence. While there is a debate if adjuvant radiation even provides a significant reduction in breast cancer-specific mortality for DCIS [88], or if any observed survival benefit should be attributed to radiotherapy’s potential systemic effects (as opposed to local disease control) [89], the impact of radiotherapy on reducing recurrence is significant. Additionally, our model identifies a low-risk group that has only an 8% 10-year risk of recurrence even without radiation. This result compares favorably to the low-risk group identified by the Oncotype DX DCIS score (10.6% 10-year recurrence risk) [90] and can suggest de-escalation/elimination of radiation therapy for this patient subgroup. Thus, our model offers distinct clinical utility for high-grade patients (who have a high recurrence risk) as well as preliminary results for low-/intermediate-grade patients. Clinically, our data has shown some potential in identifying patients who have a high risk of recurrence even after adjuvant radiotherapy. Although the sample size is very limited for this cohort, our findings provide impetus to pursue a larger study exploring this aspect. Finally, we show very preliminary results converting the final model to continuous metrics of risk which have some promise to potentially better stratify the cohorts beyond simply “high” and “low” risk. Not surprisingly, using the random forest class probabilities, from which the binary distinction is normally divided from (where the class with at least 50% trees in the random forest voting for it is chosen as the classification output), provided significant prognostic value, but has to be studied more in-depth to discern if it is a better metric rather than the binary classification that is the basis of the utilized algorithm. Unfortunately, the trained RSF continuous model, which considers the time-till event as well, seemed to not generalize as well to the validation cohort. While it did show significant prognostic value, the validation cohort had significantly higher RFS scores, wherein even the non-recurrence slides in the validation set showed much higher average scores than the training cohort recurrence slides. Potentially, this model was over-trained to the training data (and thus performed poorly on a high-grade-only cohort), was not optimally compatible with the feature selection methodology used, and/or this cohort and question was not ideal for this type of machine learning technique.

Our study has a few limitations. The first caveat is that both the training and validation cohorts originate from the same institution. Although the recurrence classifier model is “seeing” samples from patients in the validation cohort for the first time, the cohorts are likely to share some features arising from digital image generation protocols (tissue processing, staining, and imaging).

Additionally, our validation cohort consists entirely of high-grade patients. This is a potentially substantial limitation as high grade is established as a significant prognostic variable within our training cohort. Although it is important to note that finding a reliable cost-efficient prognostic variable in high-grade DCIS remains of utmost importance, as radiotherapy currently appears to be overused in high-grade DCIS compared with the reported lower recurrence rates, the value of the model in lower-grade lesions, and the view of safe radiation omission from these lower-grade patients is a valid question that has to be validated in a subsequent study.

Besides the differences in grade distributions, there exist a few other significant variations in the clinopathological and demographic variables between the two cohorts (such as necrosis and presentation), although in neither cohort are these significantly associated to the future recurrence status. Furthermore, the training cohort seemed to experience slightly higher rates of recurrence. Although training models generally perform more optimistically, the higher frequency of recurrence (positive labels) in the training cohort alongside the significant differences between classically prognostic clinopathological variables, such as grade and necrosis, might have generated a model which was less fit for the validation cohort and thus yielded lower performance in this set. Although these differences lend some credibility to the generalizability of the model, it is clear that further testing, in additional external cohorts from diverse institutions, with a variety of outcomes is required and that there might be value in retraining the model with a more thorough combined cohort.

Although our model significantly stratified patients who received radiation, in both the training and validation cohorts, the sample size is notably small and requires additional testing. Technical avenues for improvement include combining multiple image resolutions and sliding window sizes, as we had to balance the slide processing speed (20× would not be feasible to run a similar analysis on our current computers) while still preserving the structural differences that would allow pathologists to distinguish all annotated classes. An intrinsic limitation of traditional “human-crafted feature-based” ML is that feature engineering is limited to human knowledge. Alternatively, a deep learning approach, such as one involving convolutional neural networks, may be able to outperform this system and identify novel morphological signatures even more informative for patient recurrence risk prediction.

## Conclusion

The model presented in this study robustly predicts DCIS recurrence risk and significantly outperforms traditional clinicopathologic variables. Simply inputting a scan of an H&E-stained DCIS tumor slide into this tool would allow the identification of patients who are at low-risk and likely do not even require adjuvant radiation and those patients at such high risk that even more aggressive therapy may be advisable (such as systemic radiation [89]). Although this methodology is promising, it requires additional testing with more diverse samples and treatments before any clinical utility of this pipeline can be unequivocally established. Ultimately, our study provides proof of principle that such a pipeline can predict DCIS recurrence risk; in future studies, we hope to train this pipeline on images from core biopsies, as a treatment aware model, to predict patients’ recurrence risk so that their entire treatment plan (including the type of surgery and recommendations regarding radiotherapy) can be tailored based on their risk profile.

## Additional files

Additional file 1: Supplementary Methodology. (PDF 700 kb)

Additional file 2: **Supplementary Figure S1**. An example of the Graphical User Interface (GUI) developed to allow for ground truth annotations used for classifier training. Through this interface, a user will select regions representative of each class, from which the program will apply feature extraction from multiple 50x50 pixel windows within that region. (PDF 429 kb)

Additional file 3: **Supplementary Table S1**. Optical density matrix. This matrix is used to deconvolute RGB H&E images into greyscales of each layer whose intensity correlated with stain absorbance. (PDF 206 kb)

Additional file 4: **Supplementary Table S2**. Optical density matrix. This matrix is used to deconvolute RGB H&E images into greyscales of each layer whose intensity correlated with stain absorbance. (PDF 732 kb)

Additional file 5: **Supplementary Figure S2**. Summary of the methodology for model development. (A) The slide annotation classifier was developed using a random selection of slides within the training cohort. The ground truth regions were preprocessed and color deconvoluted so that texture features could be extracted from the hematoxylin distributions. Five-fold cross validation was performed to determine the model’s classification ability after which the training set was augmented through rotation and transposition of ground truth regions and input into the final annotation classifier (red box fill). (B) To develop the recurrence classifier the training slides were first annotated through the trained annotation classifier (red box fill). The fully class-annotated slides had whole slide features extracted and selected to identify the set of features that differed most significantly between patients who recurred and recurrence-free patients. The performance of these features within a classifier was determined through 5-fold cross validation, and the full training cohort was used to train a recurrence classifier (gold box fill). (C) The prognostic value of the pipeline was confirmed on a validation cohort. Both the previously-trained annotation classifier and recurrence classifier were applied towards the patient samples in this validation cohort, and the resulting stratification of patients was evaluated. (PDF 403 kb)

Additional file 6: **Supplementary Figure S3**. Example of region smoothing using a mode (class appearing most often) filter. In this example the middle tile was originally classified as a lymphocyte-dense region. The surrounding neighbors though, were predominantly classified as cancer; thus, the middle tile had its class changed to cancer. While this example showed the mode depending on each tile’s predicted class, our model actually uses the mode of tree predictions of surrounding neighbors to adjust the middle tile classification. (PDF 342 kb)

Additional file 7: **Supplementary Table 3**. Features extracted from class-annotated virtual/digital slides. The texture feature distribution statistics constitute the majority of evaluated features as they include the mean, standard deviation, skew, and kurtosis for each of the 166 textural features within each of the 5 annotated classes. (PDF 333 kb)

Additional file 8: **Supplementary Figure S4**. An example of the statistical moments obtained from full slide analysis. For each window for an annotated class the distribution of all texture features was computed. From each of these distributions, the mean, standard deviation, skew, and kurtosis was calculated and input as individual components of the full slide feature list. (PDF 285 kb)

Additional file 9: **Supplementary Equation 1**. Density Distance Statistic. Statistic comparing the size (A) and distance (D) between all (sum) cancer (i) areas (connected regions) and either immune-rich or blood vessel (BV) areas (j), normalized (divided) by the total cancer area. (PDF 296 kb)

Additional file 10: **Supplementary Figure S5**. Schematic of the logic used to translate risk category of patient slides to patient risk. Patients who possessed multiple resection slides were put into a high-risk subgroup if any of their slides were classified as high-risk by the recurrence classifier. (PDF 328 kb)

Additional file 11: **Supplementary Figure S6**. Recurrence distributions of the 159 patients in the training/test cohort, ordered according to earliest censored time or time of recurrence to last follow-up. Red points indicate a recurrence at the last follow up date while green points specify censoring. (PDF 501 kb)

Additional file 12: **Supplementary Table S4**. The distribution of baseline characteristics between patients who experienced ipsilateral recurrences versus those that did not in the training cohort. The χ2 *p* value signifies significant difference in proportions. (PDF 538 kb)

Additional file 13: **Supplementary Table S5**. Distribution of baseline characteristics between patients who experienced recurrence versus those that did not in the validation cohort. The χ2 *p*-value signifies significant difference in proportions. (PDF 538 kb)

Additional file 14: **Supplementary Figure S7**. Effect of sample size used for ground truth annotation on cross-validated accuracy. Average k-fold accuracy of annotation prediction versus number of ground truth regions. Shaded bands represent 95% confidence intervals. (PDF 229 kb)

Additional file 15: **Supplementary Table S6**. Additional confusion matrix performance metrics for the annotation classifier. (PDF 210 kb)

Additional file 16: **Supplementary Figure S8**. (A) The cumulative density function (CDF) of feature significance, noted by the t-test *p*-values, versus maximum follow-up (FU) time explored. Using 10-year recurrence, 37% of whole slidefeatures were significantly (0.05) different between patients who developed recurrence by 10 years versus those that remained recurrence-free. (B) Within this 10-year follow-up recurrence distinction, the significant feature distribution by class difference is shown in a radar plot, with the max fill (blood vessel features) indicating 39% of the filtered total significant features. (PDF 232 kb)

Additional file 17: **Supplementary Table S7**. Comparison of multiple machine learning algorithms to select the best model (and its associated features) for the recurrence classifier. ‘No annotation’ indicates the performance of a random forest model built without considering classes obtained from the first annotation step. Optimized models reflect performance after selection of optimal set of features. For each ML model, the model accuracy and high-risk group hazard ratio upon using either the full feature set or the optimized feature set, are shown. (PDF 364 kb)

Additional file 18: **Supplementary Table S8**. Feature characteristics of the final 8-feature recurrence classification model. The significance shown is based on the t-test for each feature between patients who experienced recurrence within 10 years and those that did not. The misclassification cost is computed sequentially (for e.g., the misclassification cost for feature 3 is the cost for a model which includes features 1, 2 and 3). SFTA: Segmentation-based Fractal Texture Analysis, GLRL: Grey Level Run Length, GLCO: Grey Level Co-Occurrence. (PDF 357 kb)

Additional file 19: **Supplementary Figure S9**. Combination of features produces optimal stratification. (A) Optimally stratifying patients by feature #3 provides little individual prognostic benefit. However, if patients are first split by feature #1, followed by feature #3 (B), a very significant survival difference can be observed between the high- and low-risk groups (C). (PDF 257 kb)

Additional file 20: **Supplementary Figure S10**. Kaplan-Meier curves of patients, without discordant slides, within the training cohort stratified by the trained recurrence classifier model. Significance is measured through the log-rank test. (PDF 209 kb)

Additional file 21: **Supplementary Figure S11**. Stratification of patients in training cohort using standard clinical variables. Cross validated Kaplan-Meier curves of patient outcomes (Recurrence-free survival, RFS) stratified based on (A) tumor size, (B) patient age, (C) comedo necrosis status, and (D) Nottingham grade. Significance is measured through the log-rank test. (PDF 253 kb)

Additional file 22: **Supplementary Figure S12**. (A) The Harrell’s c-statistic and 95% confidence interval for the 8-feature model and common clinopathological variables in the training cohort. (B) The Akaike Information Criterion (AIC) comparing the fit of a null model (no variables), the 8-feature model, and a model composed of the common clinopathological variables (Grade, margins status, necrosis, radiation, age, and size). The lower the AIC value the better the model fits the recurrence data. (PDF 225 kb)

Additional file 23: **Supplementary Figure S13**. Impact of clinical features on model performance when clinical variables are concatenated with the 8 features of the recurrence classifier, within a random forest model. Averaged out-of-bag feature importance (and 95% confidence intervals) from 100 models shows that clinical features do not contribute positively to the overall performance of the model. Feature importance (i.e., how heavily the model relies on each given feature for the output prediction) is defined as the change in prediction error when the values of those variables are permuted (to, in effect, break the relationship between the feature and the model outcome) across out-of-bag observations. Hence larger error changes correspond to more vital variables. Insert: Average cross-validated accuracy and hazard ratios of models built with and without clinical variables show (yes/no) significant differences. (PDF 257 kb)

Additional file 24: **Supplementary Figure S14**. Cross validated Kaplan-Meier curves of patients within the training cohort, developed by combining the testing sets for a cross validated iteration. (A) The recurrence classifier model used with Grade 3 patients’ slides only. (B) The recurrence classifier model used with Grade 1 and 2 patients’ slides only. (C) Recurrence classifier used on slides from patients who received adjuvant radiation and (D) Recurrence classifier used on slides taken from patients treated with BCS alone. (PDF 282 kb)

Additional file 25: **Supplementary Figure S15**. (A) Cross validated Kaplan-Meier curves of patients within the training cohort stratified by the trained recurrence classifier and using only invasive recurrence as an event. Significance is measured through the log-rank test. (B) Univariate and multivariate Cox regression analysis comparing the influence of common clinicopathological variables alongside the 8-feature recurrence risk prediction model for invasive recurrence-free survival, on the training set. (PDF 340 kb)

Additional file 26: **Supplementary Figure S16**. (A) Cross validated Kaplan-Meier curves of patients within the training cohort stratified by the trained recurrence classifier and using only DCIS recurrence as an event. Significance is measured through the log-rank test. (B) Univariate and multivariate Cox regression analysis comparing the influence of common clinicopathological variables alongside the 8-feature recurrence risk prediction model for DCIS recurrence-free survival, on the training set. (PDF 349 kb)

Additional file 27: **Supplementary Figure S17**. Mean values for the continuous metrics obtained when using A) the class probability, or proportion of recurrence voting trees, using the original random forest model and B) the output of a random survival forest trained with the 8 selected features. The astrix (*) represents groups with significant (*p* <0.05) differences in averages. (PDF 233 kb)

Additional file 28: **Supplementary Table S9**. Univariate cox regression analysis of the impact that continuous metrics can have on both the training (through combining the cross-validation test sets) and validation cohorts. The random survival forest (RSF) was a new model trained with the 8 selected features whereas the RF class probability reflects the continuous score obtained from counting the proportion of trees voting for ‘recurrence’ in the classification model. (PDF 399 kb)

Additional file 29: **Supplementary Figure S18**. Kaplan-Meier curves of slides within the validation cohort stratified by the trained recurrence classifier model. Significance is measured through the log-rank test. (PDF 201 kb)

Additional file 30: **Supplementary Figure S19**. Kaplan-Meier curves of patients, without discordant slides, within the validation cohort stratified by the trained recurrence classifier model. Significance is measured through the log-rank test. (PDF 199 kb)

Additional file 31: **Supplementary Fig 20**. (A) The Harrell’s c-statistic and 95% confidence interval for the 8-feature model and common clinopathological variables in the validation cohort. (B) The Akaike Information Criterion (AIC) comparing the fit of a null model (no variables), the 8-feature model, and a model composed of the common clinopathological variables (Margins status, necrosis, radiation, age, and size). The lower the AIC value the better the model fits the recurrence data. (PDF 224 kb)

Additional file 32: **Supplementary Figure S21**. Cross validated Kaplan-Meier curves of patients within the validation cohort, developed by combining the testing sets for a cross validated iteration. (A) Recurrence classifier model used on slides from patients who received adjuvant radiation and (B) Patients who were treated with BCS alone. Significance is measured through the logrank test. (PDF 221 kb)

Additional file 33: **Supplementary Figure S22**. Cross validated Kaplan-Meier curves of patients within the validation cohort, developed by combining the testing sets for a cross validated iteration. (A) Recurrence classifier model used on slides from patients who received adjuvant radiation and (B) Patients who were treated with BCS alone. Significance is measured through the logrank test. (PDF 221 kb)

Additional file 34: **Supplementary Figure S23**. (A) Kaplan-Meier curves showing robust stratification of patients in the validation cohort into high-risk of recurrence and low-risk of recurrence subgroups and using only DCIS recurrence as an event. (B) Univariate and multivariate Cox regression analysis comparing the influence of common clinicopathological variables alongside the 8-feature recurrence risk prediction model for DCIS recurrence-free survival, on the validation set. (PDF 325 kb)

## Data Availability

Data sharing is not applicable to this article as no datasets were generated or analyzed during the current study.
