# Supplementary material for: A whole slide image-based machine learning approach to predict ductal carcinoma in situ (DCIS) recurrence risk
Source: Breast Cancer Res. 2019 Jul 29;21:83. doi: 10.1186/s13058-019-1165-5 (PMC6664779; doi:10.1186/s13058-019-1165-5)
Supplement: Supplementary file 1 — Supplementary Methodology. (PDF 700 kb) [file 13058_2019_1165_MOESM1_ESM.pdf]

Supplementary Methodology

Scanning Options:

Automatic scanner mode was selected with Optovar position Pos10_1.6_1 (The Panoramic 250 Flash III, 3DHISTECH, Hungary) for good quality images scanning option (JPEG: 80, 8 bit). Flash mode was selected with 6 focus distance in field of view single layer using stitching mode without Bright-field compensation.

Time Threshold Selection:

As patients who experience recurrence after a very long follow up may possess features resembling features in patients who do not recur, it becomes imperative to select a time point that is both clinically relevant and maximizes the number of significant features that separate recurring and non-recurring groups. Therefore, t-tests were run on all of the full slide features (texture distributions, spatial features, annotation proportions, and the confidence metric) between recurrence-free and recurring (at a specified time point) patients, starting at a follow-up period of 5 years, as most patients recur within 10 years of diagnosis [1, 2]. To identify the temporal change in significant features, the same process was performed for every additional year of follow-up until a maximum follow-up period of 25 years. The maximum follow-up time selected for our study was the one which provided the greatest number of significant features between patients who recurred by that time versus those that did not.

Feature and Machine Learning Model Selection:

The full features set was first filtered to those that were significantly different (t-test p-value < 0.05) between slides of patients who recurred versus those that did not. The retained features were further evaluated by sequential forward feature selection with random forest, k-nearest neighbor, and support vector machine classifiers (Fig 1J) with the goal of identifying a classifier and a subset of features that together best predict the DCIS risk recurrence. The retained features were sequentially added one by one to the training of a classifier, and the resulting classifier's performance was measured through the misclassification rate observed upon 5-fold cross validation. Features which minimized the misclassification rate the most were retained. The process of adding features was continued until there was no further improvement in classifier's performance. The selected features alongside the classifier which provided the best cross validated accuracy and HR was selected for the final DCIS recurrence risk prediction model.

1. Donker M, Litière S, Werutsky G, Julien J-P, Fentiman IS, Agresti R, Rouanet P, de Lara CT, Bartelink H, Duez N: **Breast-conserving treatment with or without radiotherapy in ductal carcinoma in situ: 15-year recurrence rates and outcome after a recurrence, from the EORTC 10853 randomized phase III trial**. *Journal of Clinical Oncology* 2013, **31**(32):4054-4059.
2. Wärnberg F, Garmo H, Emdin S, Hedberg V, Adwall L, Sandelin K, Ringberg A, Karlsson P, Arnesson L-G, Anderson H: **Effect of radiotherapy after breast-conserving surgery for ductal carcinoma in situ: 20 years follow-up in the randomized SweDCIS trial**. *Journal of Clinical Oncology* 2014, **32**(32):3613-3618.
