# Supplementary figures and images for: A whole slide image-based machine learning approach to predict ductal carcinoma in situ (DCIS) recurrence risk
Source: Breast Cancer Res. 2019 Jul 29;21:83. doi: 10.1186/s13058-019-1165-5 (PMC6664779; doi:10.1186/s13058-019-1165-5)

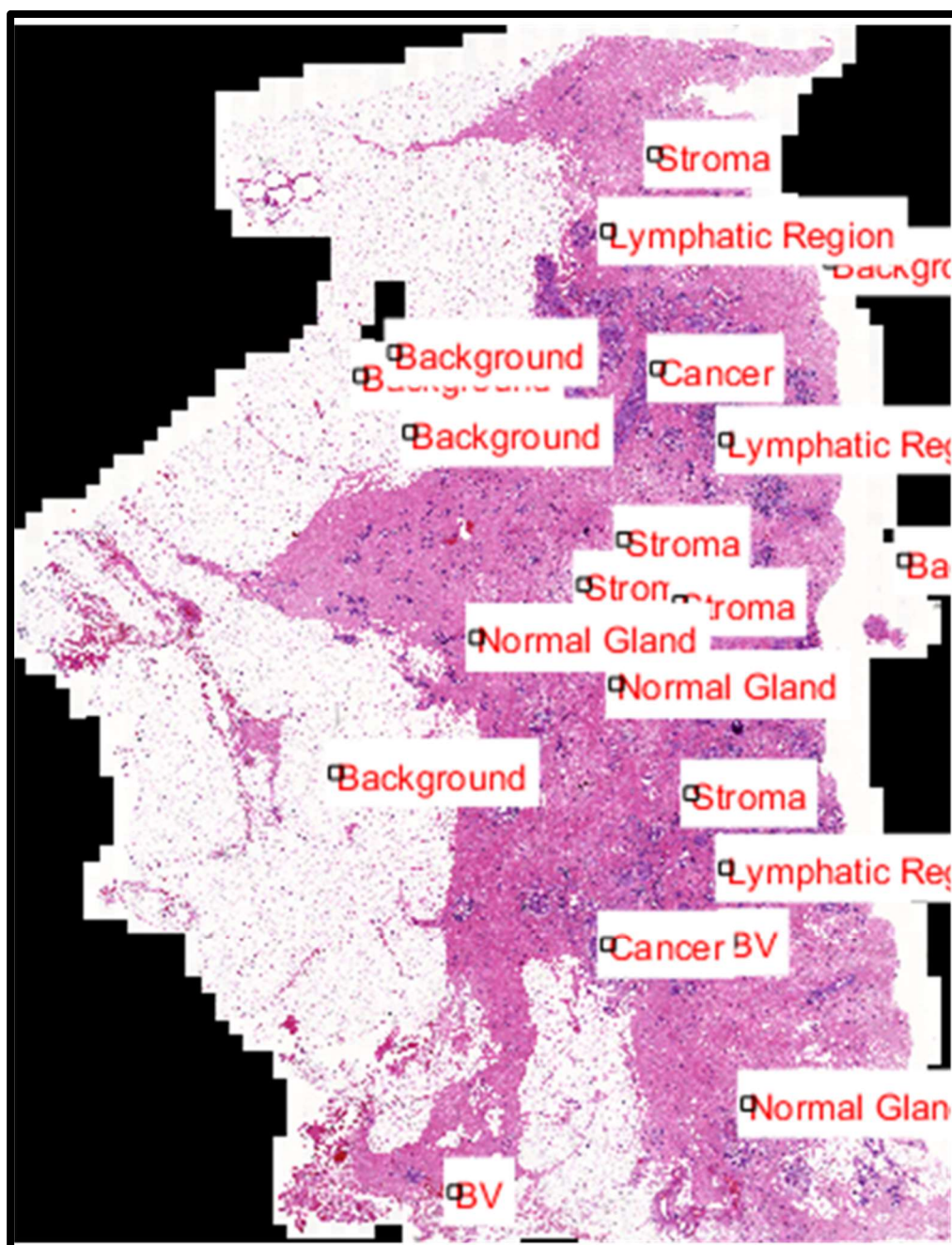

Supplement: Supplementary file 2 — Supplementary Figure S1. An example of the Graphical User Interface (GUI) developed to allow for ground truth annotations used for classifier training. Through this interface, a user will select regions representative of each class, from which the program will apply feature extraction from multiple 50x50 pixel windows within that region. (PDF 429 kb) [file 13058_2019_1165_MOESM2_ESM.pdf]

Original

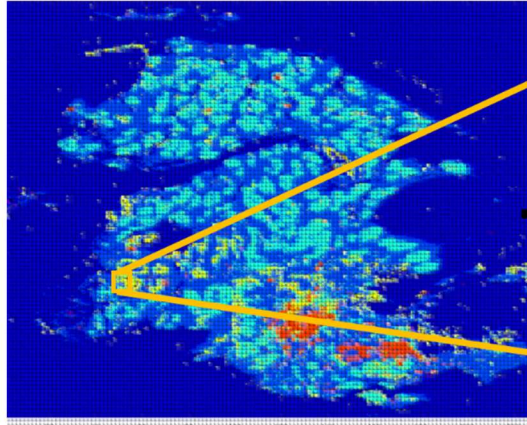

Mode Filter

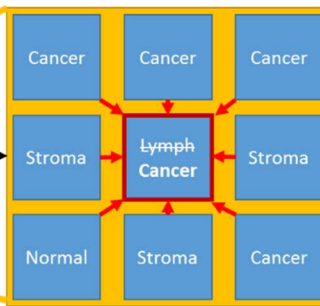

Smoothed

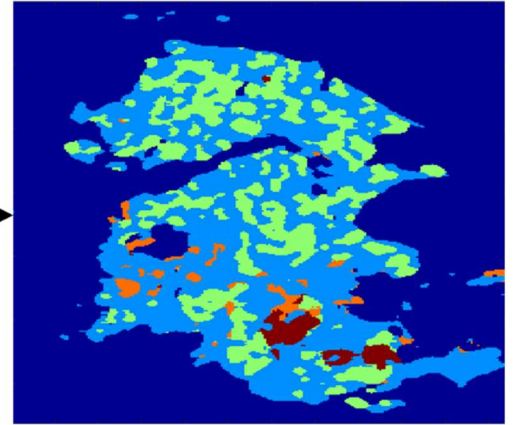

Supplement: Supplementary file 6 — Supplementary Figure S3. Example of region smoothing using a mode (class appearing most often) filter. In this example the middle tile was originally classified as a lymphocyte-dense region. The surrounding neighbors though, were predominantly classified as cancer; thus, the middle tile had its class changed to cancer. While this example showed the mode depending on each tile’s predicted class, our model actually uses the mode of tree predictions of surrounding neighbors to adjust the middle tile classification. (PDF 342 kb) [file 13058_2019_1165_MOESM6_ESM.pdf]

Single Class Annotation

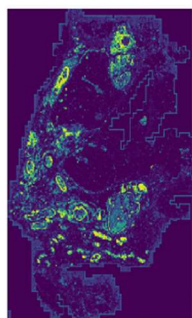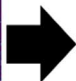

Texture Distributions

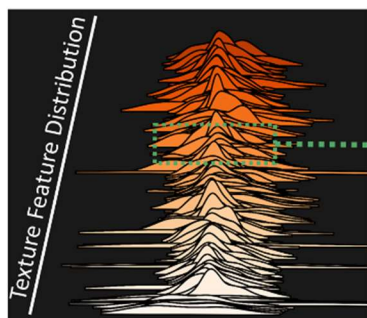

Statistical Moments per Feature

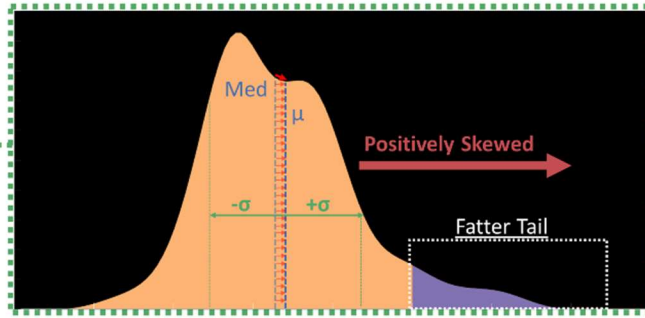

Supplement: Supplementary file 8 — Supplementary Figure S4. An example of the statistical moments obtained from full slide analysis. For each window for an annotated class the distribution of all texture features was computed. From each of these distributions, the mean, standard deviation, skew, and kurtosis was calculated and input as individual components of the full slide feature list. (PDF 285 kb) [file 13058_2019_1165_MOESM8_ESM.pdf]

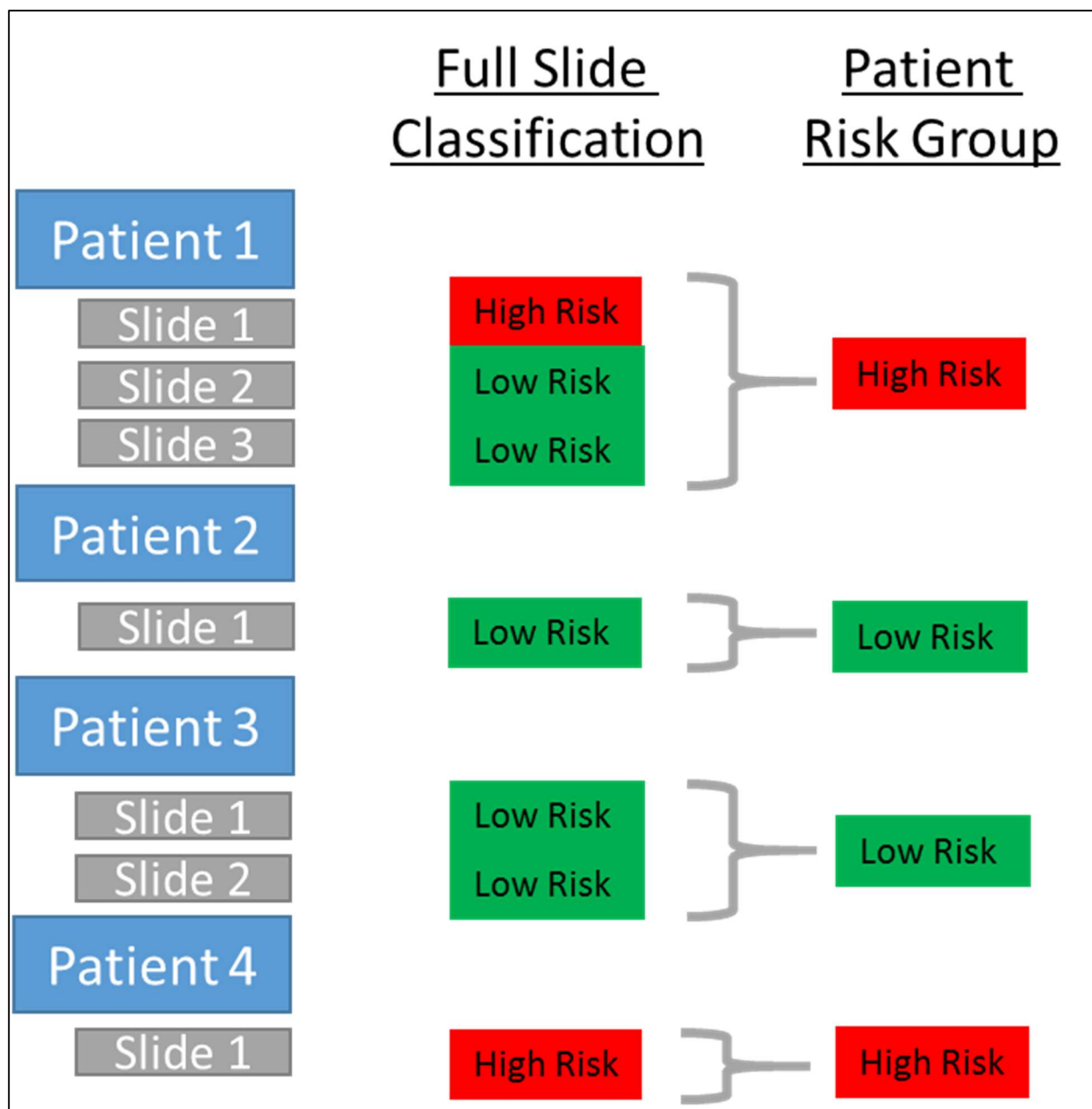

Supplement: Supplementary file 10 — Supplementary Figure S5. Schematic of the logic used to translate risk category of patient slides to patient risk. Patients who possessed multiple resection slides were put into a high-risk subgroup if any of their slides were classified as high-risk by the recurrence classifier. (PDF 328 kb) [file 13058_2019_1165_MOESM10_ESM.pdf]

Training/Test Patient Cohort

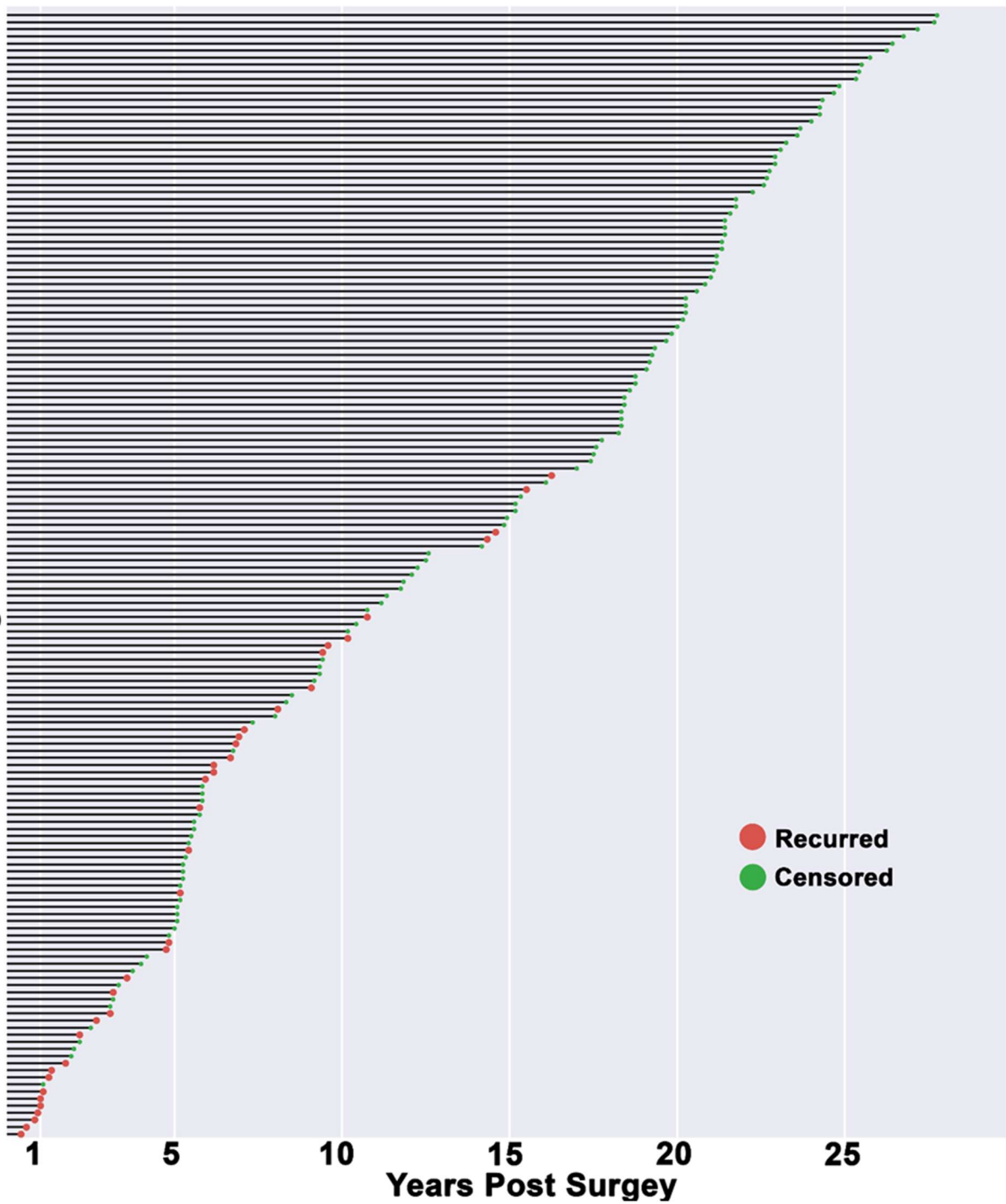

Supplement: Supplementary file 11 — Supplementary Figure S6. Recurrence distributions of the 159 patients in the training/test cohort, ordered according to earliest censored time or time of recurrence to last follow-up. Red points indicate a recurrence at the last follow up date while green points specify censoring. (PDF 501 kb) [file 13058_2019_1165_MOESM11_ESM.pdf]

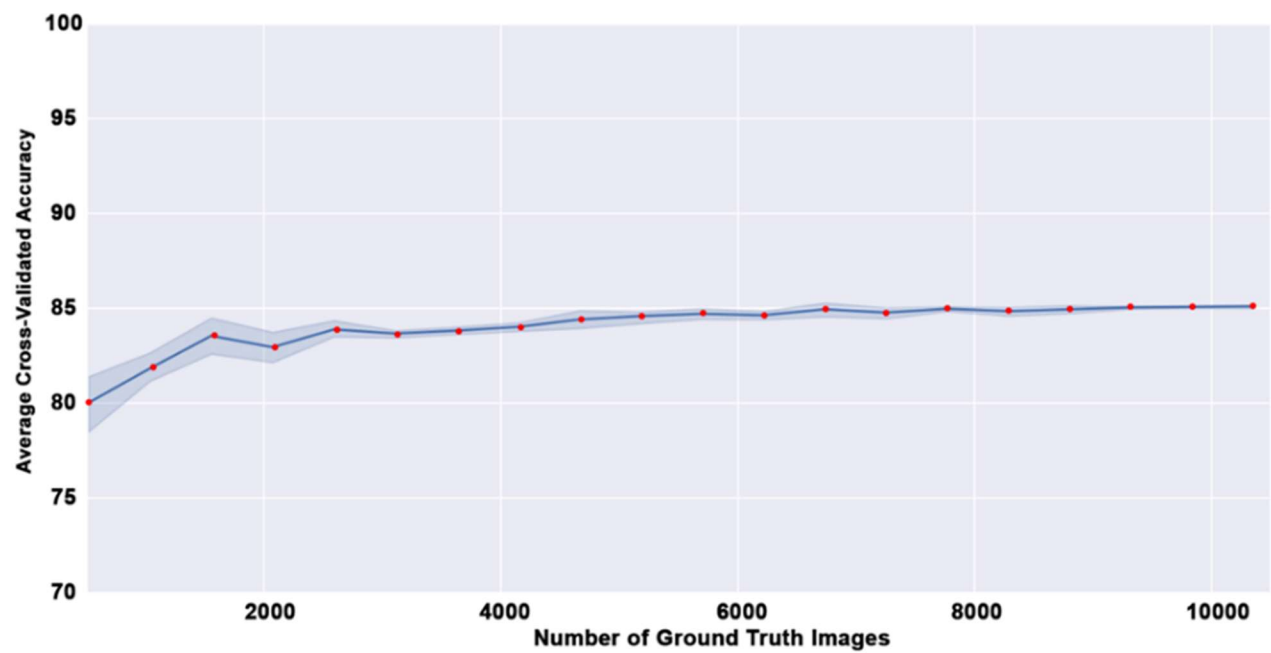

Supplement: Supplementary file 14 — Supplementary Figure S7. Effect of sample size used for ground truth annotation on cross-validated accuracy. Average k-fold accuracy of annotation prediction versus number of ground truth regions. Shaded bands represent 95% confidence intervals. (PDF 229 kb) [file 13058_2019_1165_MOESM14_ESM.pdf]

**A**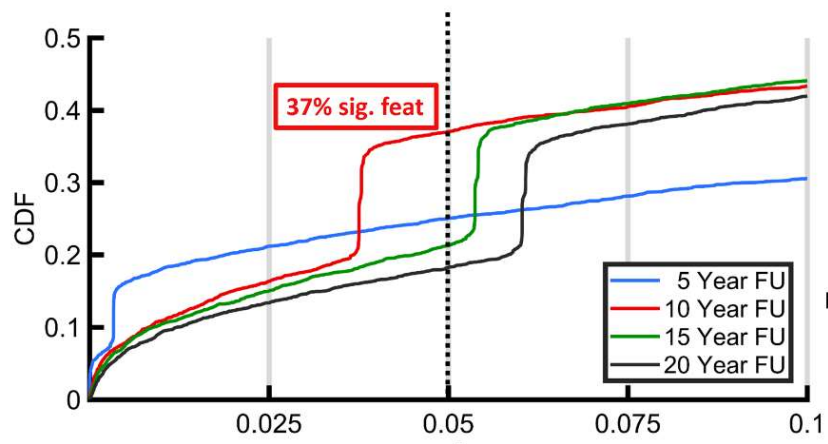**B**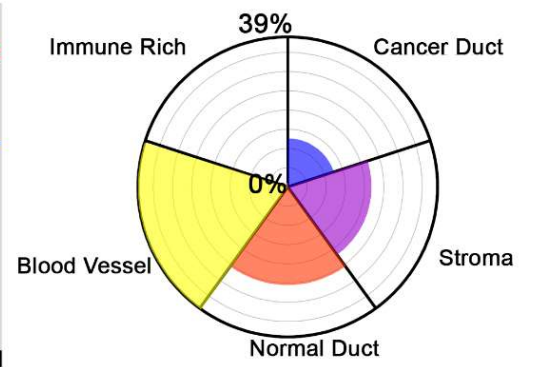

Supplement: Supplementary file 16 — Supplementary Figure S8. (A) The cumulative density function (CDF) of feature significance, noted by the t-test p-values, versus maximum follow-up (FU) time explored. Using 10-year recurrence, 37% of whole slidefeatures were significantly (0.05) different between patients who developed recurrence by 10 years versus those that remained recurrence-free. (B) Within this 10-year follow-up recurrence distinction, the significant feature distribution by class difference is shown in a radar plot, with the max fill (blood vessel features) indicating 39% of the filtered total significant features. (PDF 232 kb) [file 13058_2019_1165_MOESM16_ESM.pdf]

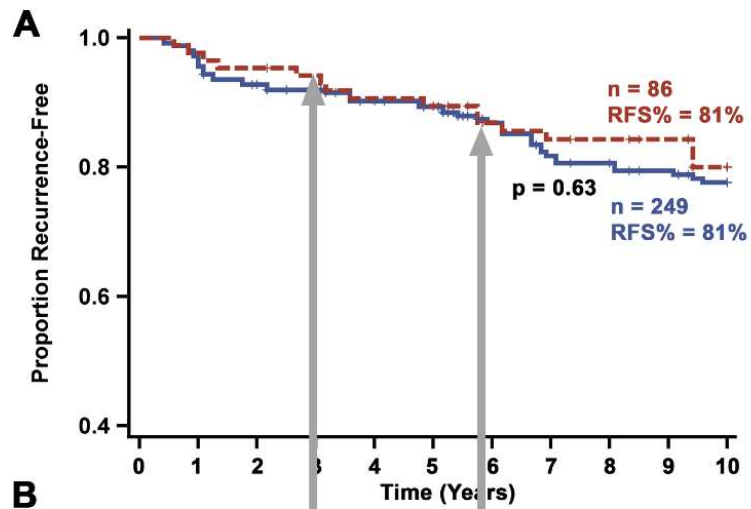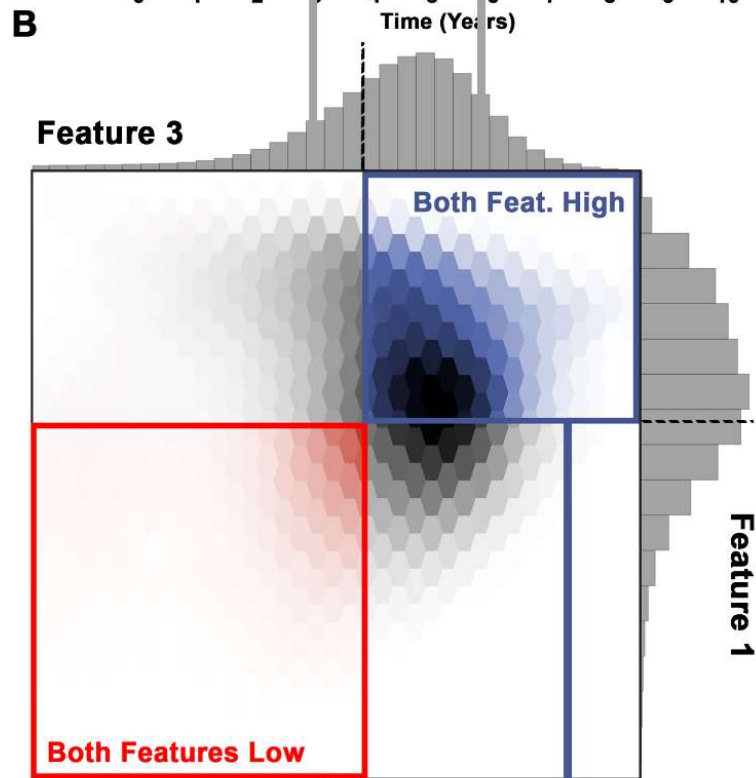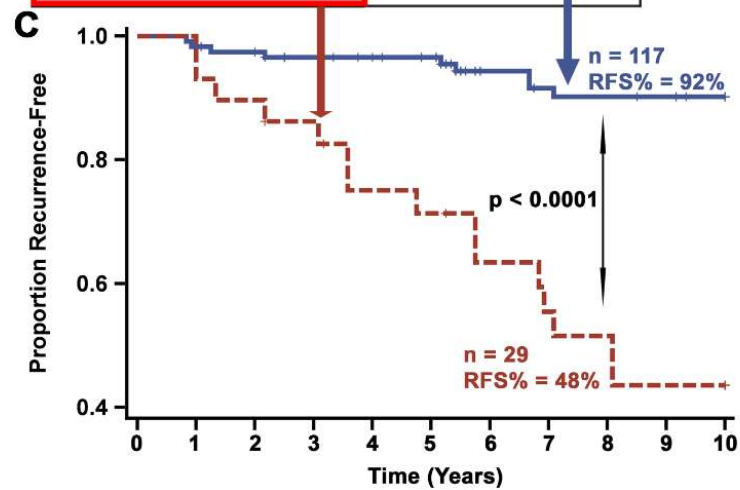

Supplement: Supplementary file 19 — Supplementary Figure S9. Combination of features produces optimal stratification. (A) Optimally stratifying patients by feature #3 provides little individual prognostic benefit. However, if patients are first split by feature #1, followed by feature #3 (B), a very significant survival difference can be observed between the high- and low-risk groups (C). (PDF 257 kb) [file 13058_2019_1165_MOESM19_ESM.pdf]

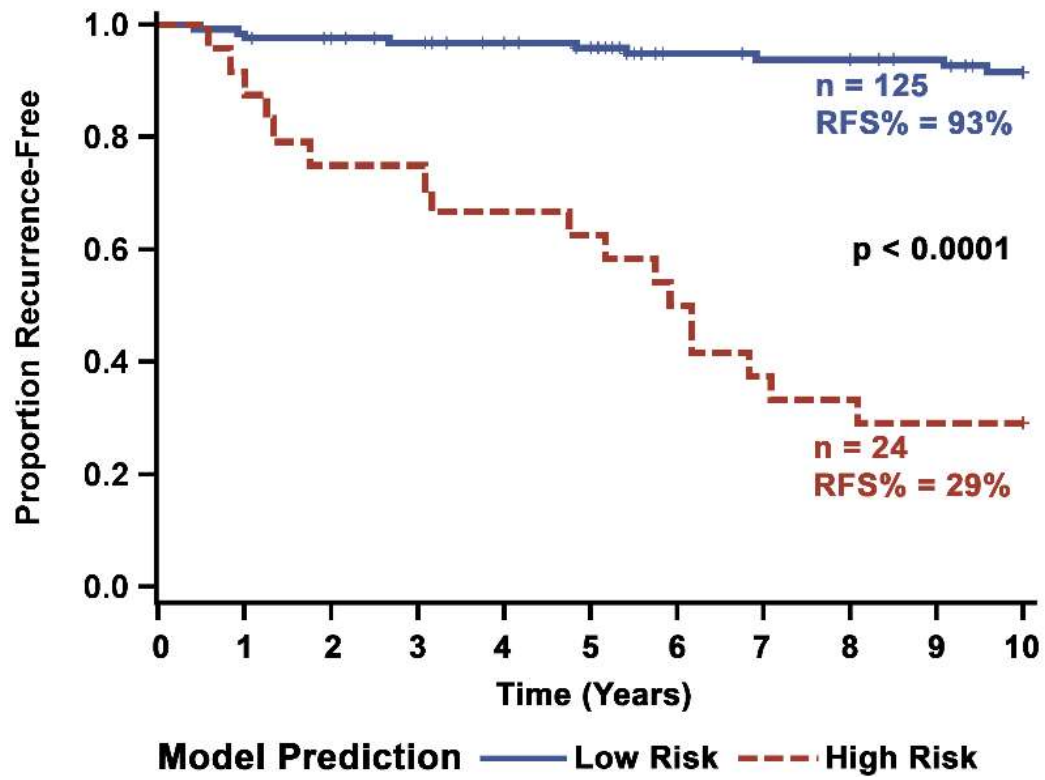

Supplement: Supplementary file 20 — Supplementary Figure S10. Kaplan-Meier curves of patients, without discordant slides, within the training cohort stratified by the trained recurrence classifier model. Significance is measured through the log-rank test. (PDF 209 kb) [file 13058_2019_1165_MOESM20_ESM.pdf]

**A**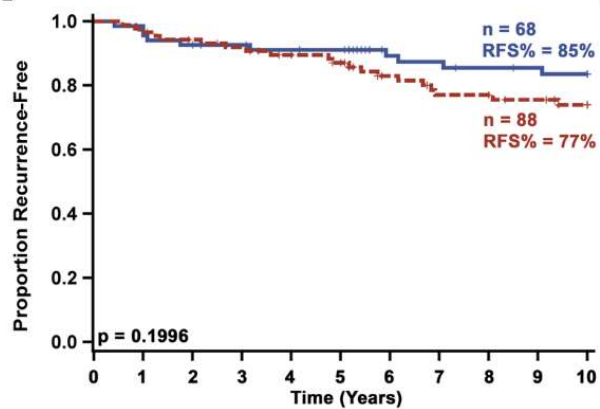**B**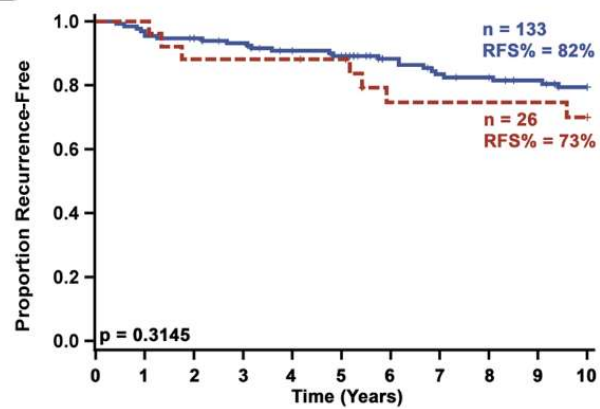**C**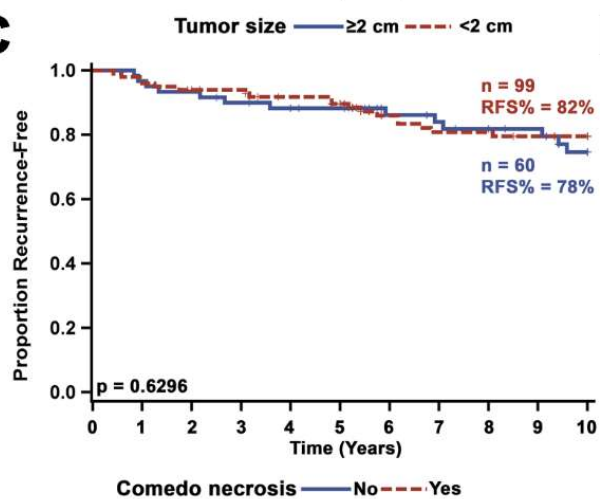**D**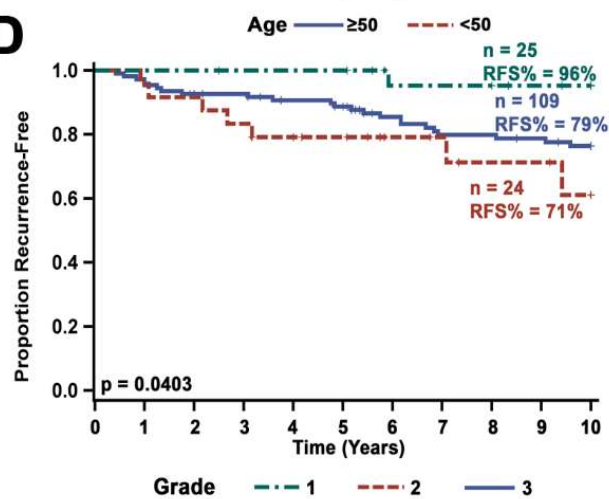

Supplement: Supplementary file 21 — Supplementary Figure S11. Stratification of patients in training cohort using standard clinical variables. Cross validated Kaplan-Meier curves of patient outcomes (Recurrence-free survival, RFS) stratified based on (A) tumor size, (B) patient age, (C) comedo necrosis status, and (D) Nottingham grade. Significance is measured through the log-rank test. (PDF 253 kb) [file 13058_2019_1165_MOESM21_ESM.pdf]

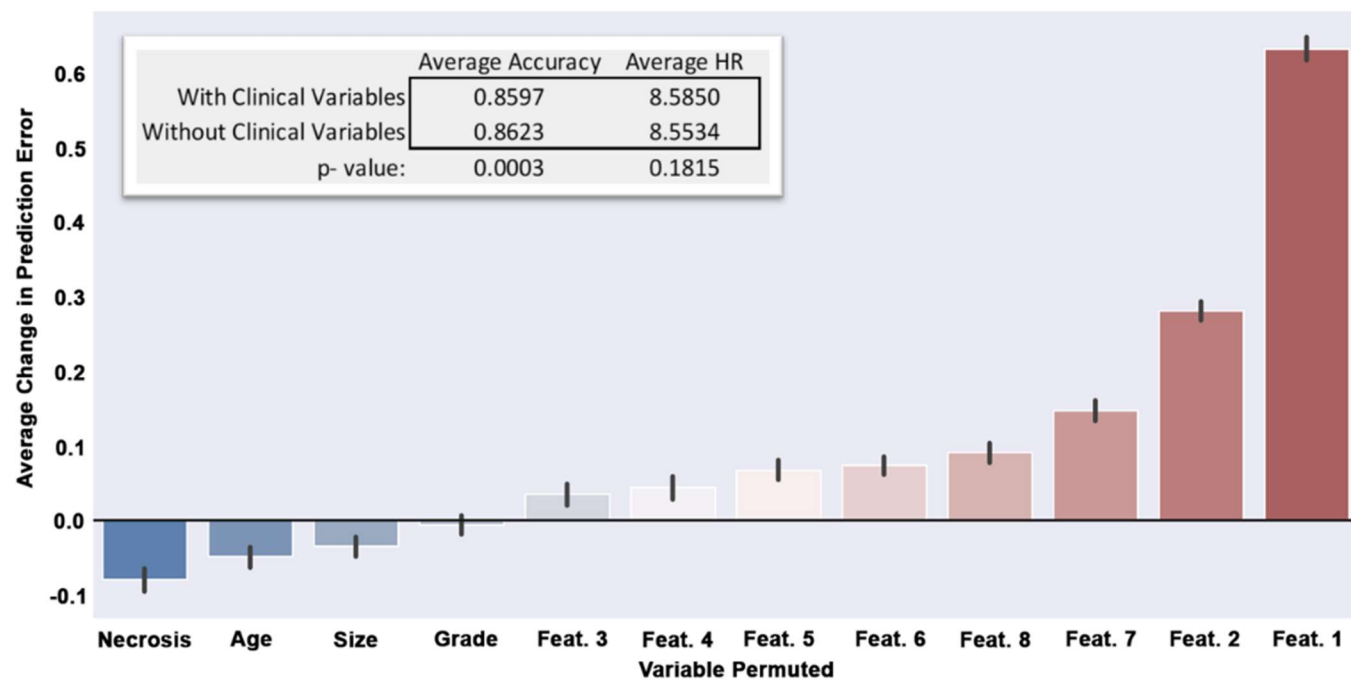

Supplement: Supplementary file 23 — Supplementary Figure S13. Impact of clinical features on model performance when clinical variables are concatenated with the 8 features of the recurrence classifier, within a random forest model. Averaged out-of-bag feature importance (and 95% confidence intervals) from 100 models shows that clinical features do not contribute positively to the overall performance of the model. Feature importance (i.e., how heavily the model relies on each given feature for the output prediction) is defined as the change in prediction error when the values of those variables are permuted (to, in effect, break the relationship between the feature and the model outcome) across out-of-bag observations. Hence larger error changes correspond to more vital variables. Insert: Average cross-validated accuracy and hazard ratios of models built with and without clinical variables show (yes/no) significant differences. (PDF 257 kb) [file 13058_2019_1165_MOESM23_ESM.pdf]

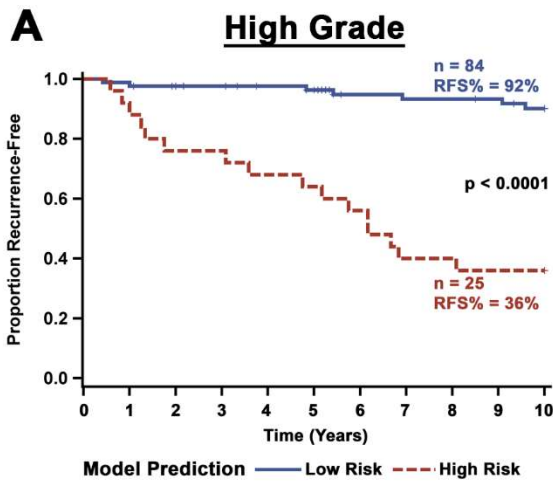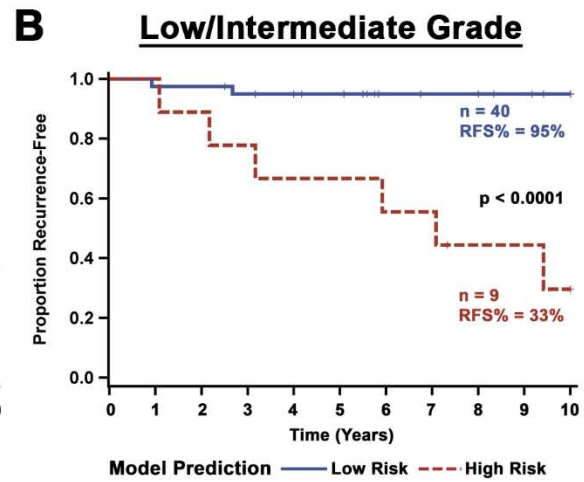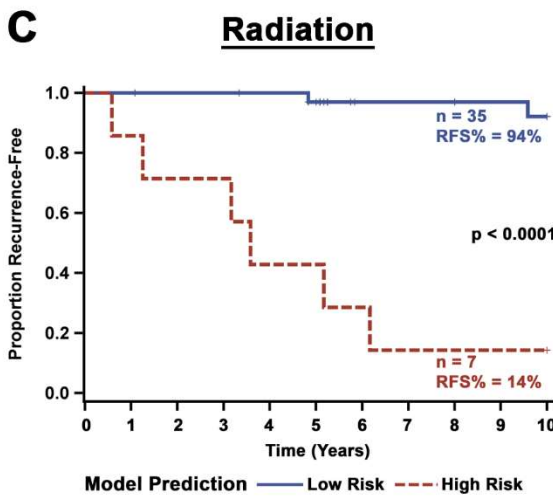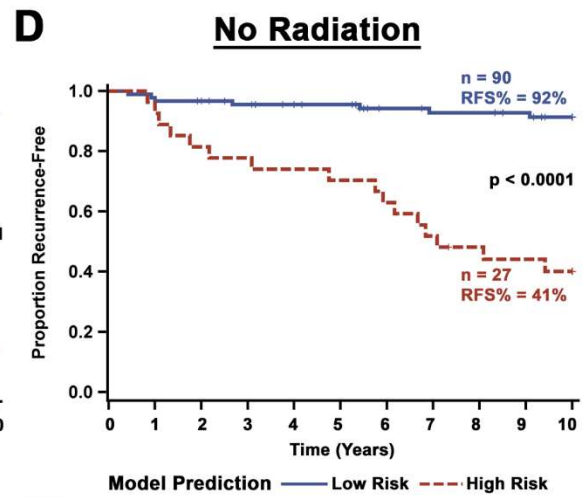

Supplement: Supplementary file 24 — Supplementary Figure S14. Cross validated Kaplan-Meier curves of patients within the training cohort, developed by combining the testing sets for a cross validated iteration. (A) The recurrence classifier model used with Grade 3 patients’ slides only. (B) The recurrence classifier model used with Grade 1 and 2 patients’ slides only. (C) Recurrence classifier used on slides from patients who received adjuvant radiation and (D) Recurrence classifier used on slides taken from patients treated with BCS alone. (PDF 282 kb) [file 13058_2019_1165_MOESM24_ESM.pdf]

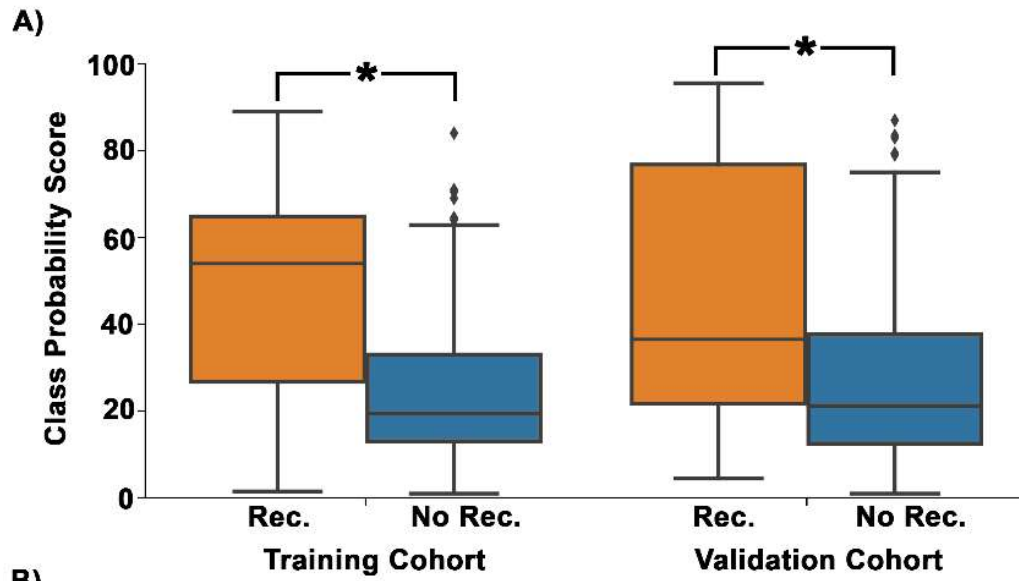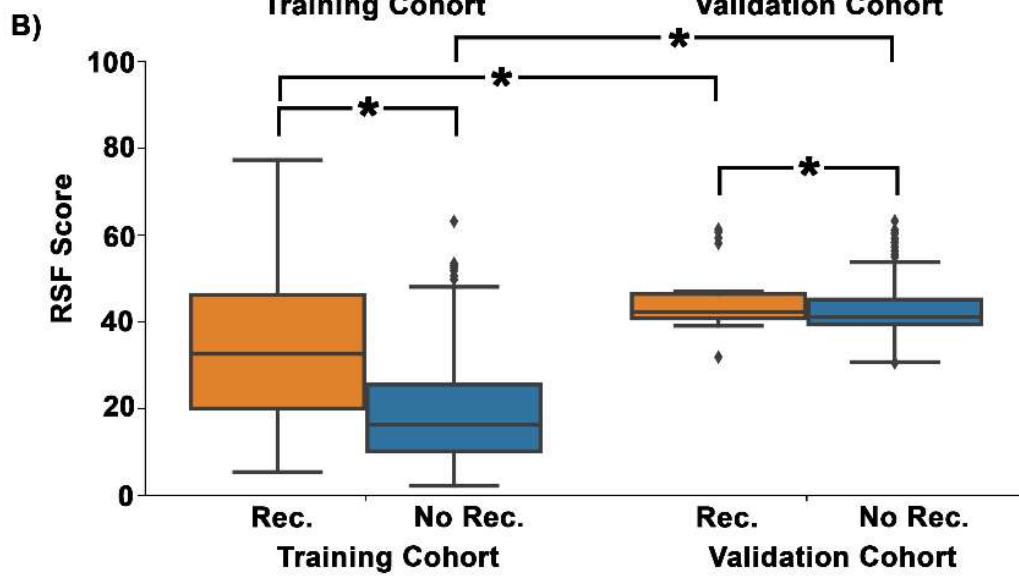

Supplement: Supplementary file 27 — Supplementary Figure S17. Mean values for the continuous metrics obtained when using A) the class probability, or proportion of recurrence voting trees, using the original random forest model and B) the output of a random survival forest trained with the 8 selected features. The astrix (*) represents groups with significant (p <0.05) differences in averages. (PDF 233 kb) [file 13058_2019_1165_MOESM27_ESM.pdf]

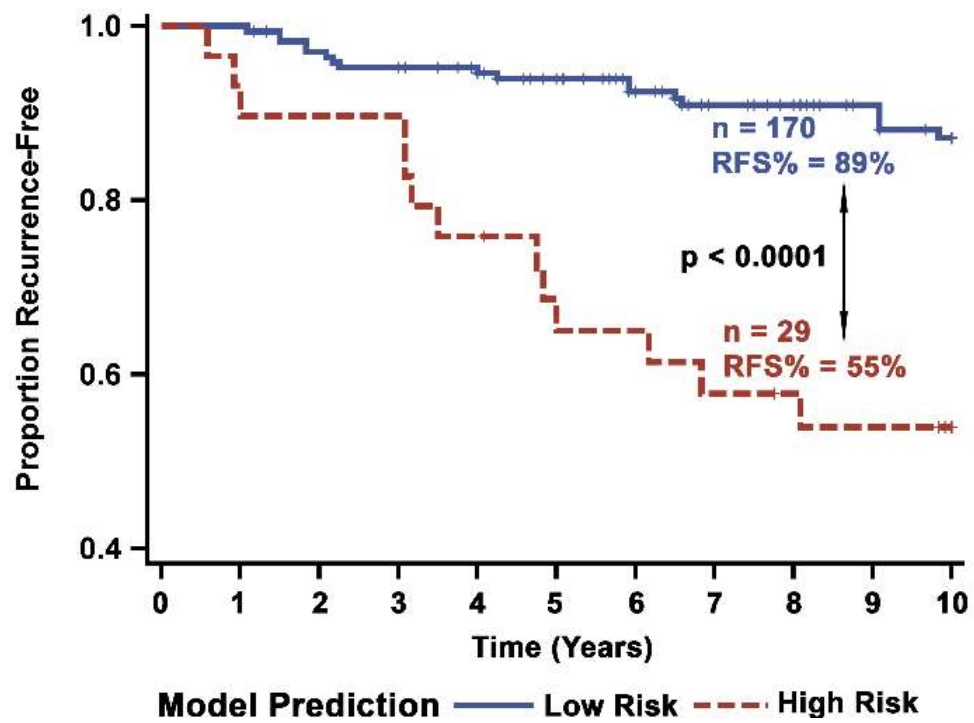

Supplement: Supplementary file 29 — Supplementary Figure S18. Kaplan-Meier curves of slides within the validation cohort stratified by the trained recurrence classifier model. Significance is measured through the log-rank test. (PDF 201 kb) [file 13058_2019_1165_MOESM29_ESM.pdf]

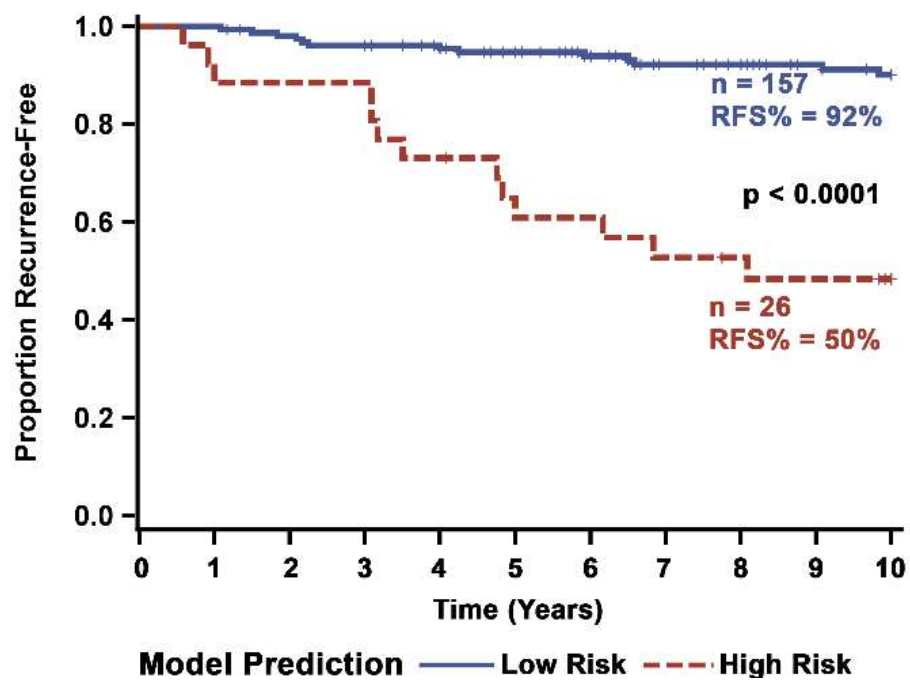

Supplement: Supplementary file 30 — Supplementary Figure S19. Kaplan-Meier curves of patients, without discordant slides, within the validation cohort stratified by the trained recurrence classifier model. Significance is measured through the log-rank test. (PDF 199 kb) [file 13058_2019_1165_MOESM30_ESM.pdf]

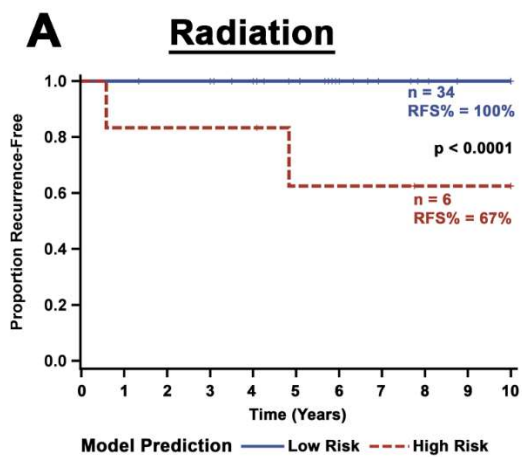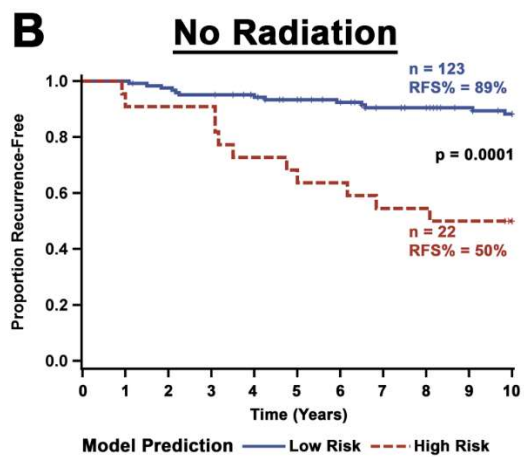

Supplement: Supplementary file 32 — Supplementary Figure S21. Cross validated Kaplan-Meier curves of patients within the validation cohort, developed by combining the testing sets for a cross validated iteration. (A) Recurrence classifier model used on slides from patients who received adjuvant radiation and (B) Patients who were treated with BCS alone. Significance is measured through the logrank test. (PDF 221 kb) [file 13058_2019_1165_MOESM32_ESM.pdf]
