# Supplementary material for: A whole slide image-based machine learning approach to predict ductal carcinoma in situ (DCIS) recurrence risk
Source: Breast Cancer Res. 2019 Jul 29;21:83. doi: 10.1186/s13058-019-1165-5 (PMC6664779; doi:10.1186/s13058-019-1165-5)
Supplement: Supplementary file 3 — Supplementary Table S1. Optical density matrix. This matrix is used to deconvolute RGB H&E images into greyscales of each layer whose intensity correlated with stain absorbance. (PDF 206 kb) [file 13058_2019_1165_MOESM3_ESM.pdf]

	R	G	B
Hematoxylin	0.644	0.717	0.267
Eosin	0.093	0.954	0.283
Zero Matrix	0.636	0.001	0.772
