# Supplementary material for: A whole slide image-based machine learning approach to predict ductal carcinoma in situ (DCIS) recurrence risk
Source: Breast Cancer Res. 2019 Jul 29;21:83. doi: 10.1186/s13058-019-1165-5 (PMC6664779; doi:10.1186/s13058-019-1165-5)
Supplement: Supplementary file 4 — Supplementary Table S2. Optical density matrix. This matrix is used to deconvolute RGB H&E images into greyscales of each layer whose intensity correlated with stain absorbance. (PDF 732 kb) [file 13058_2019_1165_MOESM4_ESM.pdf]

Textural Feature Type	No. of Features	Source
Entropy	1	[1]
Gray-Level Co-occurrence Matrix (GLCO)	16	[2]
Gray-Level Run Length (GRLRL)	44	[3, 4]
Segmentation-based Fractal Texture Analysis (STFA)	45	[5, 6]
Gabor wavelet filters	60	[7-10]
Sum:	166	

1. Gonzalez RC, R.E. Woods, S.L. Eddins: **Chapter 11**. In: *Digital Image Processing Using MATLAB.* edn. New Jersey: Prentice Hall; 2003.
2. Haralick RM, Shanmugam K: **Textural features for image classification**. *IEEE Transactions on systems, man, and cybernetics* 1973, **3**(6):610-621.
3. Galloway MM: **Texture analysis using gray level run lengths**. *Computer Graphics and Image Processing* 1975, **4**(2):172-179.
4. Wei X: **Gray Level Run Length Matrix Toolbox v1.0**. In.; 2007.
5. Costa A: **alceufc/sfta**. In. https://www.mathworks.com/matlabcentral/fileexchange/37933-alceufc-sfta: MATLAB Central File Exchange; 11/2/2016.
6. Costa AF, Humpire-Mamani G, Traina AJM: **An efficient algorithm for fractal analysis of textures**. In: *Graphics, Patterns and Images (SIBGRAPI), 2012 25th SIBGRAPI Conference on: 2012*: IEEE; 2012: 39-46.
7. Kuse M: **Gabor Image Features**. In. http://www.mathworks.com/matlabcentral/fileexchange/38844-gabor-image-features?focused=5249516&tab=function: MATLAB Central File Exchange; 10/30/2012.
8. Kovesi P: **Symmetry and asymmetry from local phase**. In: *Tenth Australian joint conference on artificial intelligence: 1997*: Citeseer; 1997: 2-4.
9. Kovesi P: **Image features from phase congruency**. *Videre: Journal of computer vision research* 1999, **1**(3):1-26.
10. Kuse M, Wang Y-F, Kalasannavar V, Khan M, Rajpoot N: **Local isotropic phase symmetry measure for detection of beta cells and lymphocytes**. *Journal of pathology informatics* 2011, **2**.
