# Supplementary material for: A whole slide image-based machine learning approach to predict ductal carcinoma in situ (DCIS) recurrence risk
Source: Breast Cancer Res. 2019 Jul 29;21:83. doi: 10.1186/s13058-019-1165-5 (PMC6664779; doi:10.1186/s13058-019-1165-5)
Supplement: Supplementary file 5 — Supplementary Figure S2. Summary of the methodology for model development. (A) The slide annotation classifier was developed using a random selection of slides within the training cohort. The ground truth regions were preprocessed and color deconvoluted so that texture features could be extracted from the hematoxylin distributions. Five-fold cross validation was performed to determine the model’s classification ability after which the training set was augmented through rotation and transposition of ground truth regions and input into the final annotation classifier (red box fill). (B) To develop the recurrence classifier the training slides were first annotated through the trained annotation classifier (red box fill). The fully class-annotated slides had whole slide features extracted and selected to identify the set of features that differed most significantly between patients who recurred and recurrence-free patients. The performance of these features within a classifier was determined through 5-fold cross validation, and the full training cohort was used to train a recurrence classifier (gold box fill). (C) The prognostic value of the pipeline was confirmed on a validation cohort. Both the previously-trained annotation classifier and recurrence classifier were applied towards the patient samples in this validation cohort, and the resulting stratification of patients was evaluated. (PDF 403 kb) [file 13058_2019_1165_MOESM5_ESM.pdf]

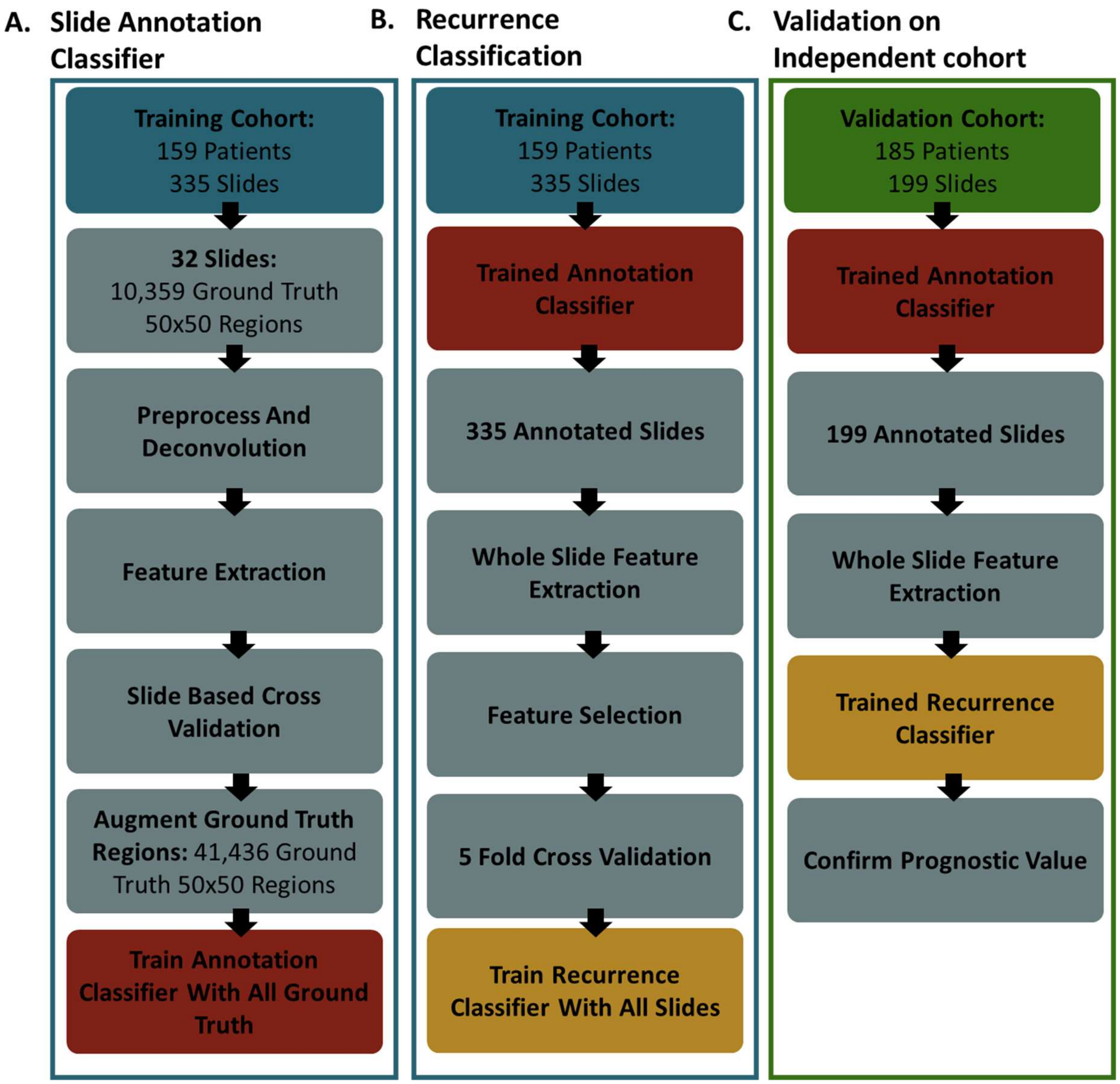
A. Slide Annotation Classifier
Training Cohort: 159 Patients 335 Slides
32 Slides: 10,359 Ground Truth 50x50 Regions
Preprocess And Deconvolution
Feature Extraction
Slide Based Cross Validation
Augment Ground Truth Regions: 41,436 Ground Truth 50x50 Regions
Train Annotation Classifier With All Ground Truth
B. Recurrence Classification
Training Cohort: 159 Patients 335 Slides
Trained Annotation Classifier
335 Annotated Slides
Whole Slide Feature Extraction
Feature Selection
5 Fold Cross Validation
Train Recurrence Classifier With All Slides
C. Validation on Independent cohort
Validation Cohort: 185 Patients 199 Slides
Trained Annotation Classifier
199 Annotated Slides
Whole Slide Feature Extraction
Trained Recurrence Classifier
Confirm Prognostic Value
