# Supplementary material for: A whole slide image-based machine learning approach to predict ductal carcinoma in situ (DCIS) recurrence risk
Source: Breast Cancer Res. 2019 Jul 29;21:83. doi: 10.1186/s13058-019-1165-5 (PMC6664779; doi:10.1186/s13058-019-1165-5)
Supplement: Supplementary file 7 — Supplementary Table 3. Features extracted from class-annotated virtual/digital slides. The texture feature distribution statistics constitute the majority of evaluated features as they include the mean, standard deviation, skew, and kurtosis for each of the 166 textural features within each of the 5 annotated classes. (PDF 333 kb) [file 13058_2019_1165_MOESM7_ESM.pdf]

Full-Slide Feature Type	No. of Features
Distribution Statistics	3320
Spatial Distance Densities	12
Class Proportions	5
Confidence Metric	5
