# Supplementary material for: A whole slide image-based machine learning approach to predict ductal carcinoma in situ (DCIS) recurrence risk
Source: Breast Cancer Res. 2019 Jul 29;21:83. doi: 10.1186/s13058-019-1165-5 (PMC6664779; doi:10.1186/s13058-019-1165-5)
Supplement: Supplementary file 9 — Supplementary Equation 1. Density Distance Statistic. Statistic comparing the size (A) and distance (D) between all (sum) cancer (i) areas (connected regions) and either immune-rich or blood vessel (BV) areas (j), normalized (divided) by the total cancer area. (PDF 296 kb) [file 13058_2019_1165_MOESM9_ESM.pdf]

$$Density\ Distance = \frac{\sum_{i=1}^{n} \sum_{j=1}^{n} \frac{1}{D_{i,j}} * A_i * A_j}{\sum A_i}$$
