# Supplementary material for: A whole slide image-based machine learning approach to predict ductal carcinoma in situ (DCIS) recurrence risk
Source: Breast Cancer Res. 2019 Jul 29;21:83. doi: 10.1186/s13058-019-1165-5 (PMC6664779; doi:10.1186/s13058-019-1165-5)
Supplement: Supplementary file 12 — Supplementary Table S4. The distribution of baseline characteristics between patients who experienced ipsilateral recurrences versus those that did not in the training cohort. The χ2 p value signifies significant difference in proportions. (PDF 538 kb) [file 13058_2019_1165_MOESM12_ESM.pdf]

Training Cohort Clinical Characteristics by 10 Year Recurrence Status			
**Baseline characteristic**	**Recurred (N = 31)**	**Rec. Free (N = 128)**	**p value**
**Patient age**			
Median Age (range), years	55 (41 - 73)	57 (30 - 83)	0.3119
Age <50, n (%)	7 (22.6)	19 (14.8)	
Age>=50, n (%)	24 (77.4)	109 (85.2)	
**Menopausal Status, n (%)**			
Pre	8 (25.8)	23 (18.0)	0.3359
Post	23 (74.2)	105 (82.0)	
**Presentation, n (%)**			
Screening	12 (38.7)	73 (57.0)	0.0663
Symptomatic	19 (61.3)	55 (43.0)	
**Comedo Necrosis, n (%)**			
No	13 (41.9)	47 (36.7)	0.5927
Yes	18 (58.1)	81 (63.3)	
**Radiation, n (%)**			
No	23 (74.2)	94 (73.4)	0.9316
Yes	8 (25.8)	34 (26.6)	
**Grade, n (%)**			
1	1 (3.2)	24 (18.9)	0.0343
2	7 (22.6)	17 (13.4)	
3	23 (74.2)	86 (67.7)	
**Margins, n (%)**			
Negative	30 (96.8)	124 (97.6)	0.79
Positive	1 (3.2)	3 (2.4)	
**Tumor Size**			
Median Tumor Size	1.5 (0.4 - 5.0)	1.8 (0.1 - 14.5)	0.2029
Size <2.0, n (%)	20 (66.7)	68 (54.0)	
Size >=2.0, n (%)	10 (33.3)	58 (46.0)	
