# Supplementary material for: A whole slide image-based machine learning approach to predict ductal carcinoma in situ (DCIS) recurrence risk
Source: Breast Cancer Res. 2019 Jul 29;21:83. doi: 10.1186/s13058-019-1165-5 (PMC6664779; doi:10.1186/s13058-019-1165-5)
Supplement: Supplementary file 13 — Supplementary Table S5. Distribution of baseline characteristics between patients who experienced recurrence versus those that did not in the validation cohort. The χ2 p-value signifies significant difference in proportions. (PDF 538 kb) [file 13058_2019_1165_MOESM13_ESM.pdf]

Validation Cohort Clinical Characteristics by 10 Year Recurrence Status			
**Baseline characteristic**	**Recurred (N = 26)**	**Rec. Free (N = 159)**	**p value**
**Patient age**			
Median Age (range), years	62 (44 - 73)	59 (36 - 77)	0.404
Age <50, n (%)	2 (7.7)	21 (13.2)	
Age>=50, n (%)	24 (92.3)	138 (86.8)	
**Menopausal Status, n (%)**			
Pre	4 (15.4)	25 (15.7)	0.9648
Post	22 (84.6)	134 (84.3)	
**Presentation, n (%)**			
Screening	16 (61.5)	104 (65.4)	0.7031
Symptomatic	10 (38.5)	55 (34.6)	
**Comedo Necrosis, n (%)**			
No	6 (23.1)	28 (17.6)	0.5151
Yes	20 (76.9)	131 (82.4)	
**Radiation, n (%)**			
No	24 (92.3)	121 (76.1)	0.0406
Yes	2 (7.7)	38 (23.9)	
**Grade, n (%)**			
1	0 (0)	0 (0)	-
2	0 (0)	0 (0)	
3	26 (100)	159 (100)	
**Margins, n (%)**			
Negative	26 (100)	157 (98.7)	0.435
Positive	0 (0)	2 (1.3)	
**Tumor Size**			
Median Tumor Size	1.5 (0.2 - 12.0)	1.8 (0.3 - 11.0)	0.1009
Size <2.0, n (%)	18 (69.2)	83 (52.2)	
Size >=2.5, n (%)	8 (30.8)	76 (47.8)	
