# Supplementary material for: A whole slide image-based machine learning approach to predict ductal carcinoma in situ (DCIS) recurrence risk
Source: Breast Cancer Res. 2019 Jul 29;21:83. doi: 10.1186/s13058-019-1165-5 (PMC6664779; doi:10.1186/s13058-019-1165-5)
Supplement: Supplementary file 15 — Supplementary Table S6. Additional confusion matrix performance metrics for the annotation classifier. (PDF 210 kb) [file 13058_2019_1165_MOESM15_ESM.pdf]

| Annotation | Sensitivity | Specificity | Precision | Recall | F-Score |
| --- | --- | --- | --- | --- | --- |
| Background | 0.96 | 1.00 | 0.96 | 0.96 | 0.96 |
| Stroma | 0.85 | 0.97 | 0.92 | 0.85 | 0.88 |
| Benign Ducts | 0.76 | 0.94 | 0.67 | 0.76 | 0.71 |
| Cancer Duct | 0.85 | 0.89 | 0.86 | 0.85 | 0.86 |
| Immune Rich | 0.91 | 0.99 | 0.77 | 0.91 | 0.83 |
| Blood Vessel | 0.91 | 0.99 | 0.71 | 0.91 | 0.79 |
