# Supplementary material for: A whole slide image-based machine learning approach to predict ductal carcinoma in situ (DCIS) recurrence risk
Source: Breast Cancer Res. 2019 Jul 29;21:83. doi: 10.1186/s13058-019-1165-5 (PMC6664779; doi:10.1186/s13058-019-1165-5)
Supplement: Supplementary file 17 — Supplementary Table S7. Comparison of multiple machine learning algorithms to select the best model (and its associated features) for the recurrence classifier. ‘No annotation’ indicates the performance of a random forest model built without considering classes obtained from the first annotation step. Optimized models reflect performance after selection of optimal set of features. For each ML model, the model accuracy and high-risk group hazard ratio upon using either the full feature set or the optimized feature set, are shown. (PDF 364 kb) [file 13058_2019_1165_MOESM17_ESM.pdf]

Model	Average Accuracy (std.)	Average Hazard Ratio (std.)
Optimized Random Forest (RF)	**0.86 (0.010)**	**8.55 (1.272)**
Full Feature Non Annotated RF	0.78 (0.012)	3.08 (0.496)
Optimized Non Annotated RF	0.79 (0.008)	2.82 (0.283)
Full Feature SVM Model	0.80 (0.002)	1.21 (0.174)
Optimized SVM Model	0.80 (0.037)	7.27 (4.537)
Full Feature KNN Model	0.79 (0.004)	1.59 (0.347)
Optimized KNN Model	0.80 (0.009)	4.31 (0.537)
