# Supplementary material for: A whole slide image-based machine learning approach to predict ductal carcinoma in situ (DCIS) recurrence risk
Source: Breast Cancer Res. 2019 Jul 29;21:83. doi: 10.1186/s13058-019-1165-5 (PMC6664779; doi:10.1186/s13058-019-1165-5)
Supplement: Supplementary file 18 — Supplementary Table S8. Feature characteristics of the final 8-feature recurrence classification model. The significance shown is based on the t-test for each feature between patients who experienced recurrence within 10 years and those that did not. The misclassification cost is computed sequentially (for e.g., the misclassification cost for feature 3 is the cost for a model which includes features 1, 2 and 3). SFTA: Segmentation-based Fractal Texture Analysis, GLRL: Grey Level Run Length, GLCO: Grey Level Co-Occurrence. (PDF 357 kb) [file 13058_2019_1165_MOESM18_ESM.pdf]

Feature Information	Features Added							
	Feat. 1	Feat. 2	Feat. 3	Feat. 4	Feat. 5	Feat. 6	Feat. 7	Feat. 8
**Significance (p value)**	0	0.0146	0.0151	0.0376	0.008	0.0041	0.0014	0.0146
**Misclassification Cost After Add**	0.256	0.194	0.167	0.14	0.128	0.125	0.113	0.101
**General Category**	STFA	STFA	GLRL	GLRL	Gabor	GLCO	Gabor	GLCO
**Statistical Moment**	Mean	SD	Mean	Mean	Skew	Kurt.	Skew	Skew
**Tissue Annotation**	Cancer	Stroma	Cancer	Normal	Cancer	Cancer	Stroma	BV
**Overexpressed In**	No Rec.	Rec.	No Rec.	Rec.	No Rec.	Rec.	No Rec.	Rec.
