# Supplementary material for: A whole slide image-based machine learning approach to predict ductal carcinoma in situ (DCIS) recurrence risk
Source: Breast Cancer Res. 2019 Jul 29;21:83. doi: 10.1186/s13058-019-1165-5 (PMC6664779; doi:10.1186/s13058-019-1165-5)
Supplement: Supplementary file 22 — Supplementary Figure S12. (A) The Harrell’s c-statistic and 95% confidence interval for the 8-feature model and common clinopathological variables in the training cohort. (B) The Akaike Information Criterion (AIC) comparing the fit of a null model (no variables), the 8-feature model, and a model composed of the common clinopathological variables (Grade, margins status, necrosis, radiation, age, and size). The lower the AIC value the better the model fits the recurrence data. (PDF 225 kb) [file 13058_2019_1165_MOESM22_ESM.pdf]

A

Variable	Harrell's c-statistic (95% CI)
Predictive Model	**0.77 (0.69 - 0.85)**
Grade	0.52 (0.44 - 0.60)
Margin Status	0.50 (0.48 - 0.52)
Necrosis	0.52 (0.43 - 0.60)
Radiation	0.51 (0.43 - 0.58)
Age	0.56 (0.46 - 0.67)
Size	0.57 (0.48 - 0.67)

B

Model	Akaike Information Criterion
Null Model	278.33
Predictive Model	**239.80**
Clinopathological Variables	276.93
