# Supplementary material for: A whole slide image-based machine learning approach to predict ductal carcinoma in situ (DCIS) recurrence risk
Source: Breast Cancer Res. 2019 Jul 29;21:83. doi: 10.1186/s13058-019-1165-5 (PMC6664779; doi:10.1186/s13058-019-1165-5)
Supplement: Supplementary file 25 — Supplementary Figure S15. (A) Cross validated Kaplan-Meier curves of patients within the training cohort stratified by the trained recurrence classifier and using only invasive recurrence as an event. Significance is measured through the log-rank test. (B) Univariate and multivariate Cox regression analysis comparing the influence of common clinicopathological variables alongside the 8-feature recurrence risk prediction model for invasive recurrence-free survival, on the training set. (PDF 340 kb) [file 13058_2019_1165_MOESM25_ESM.pdf]

A

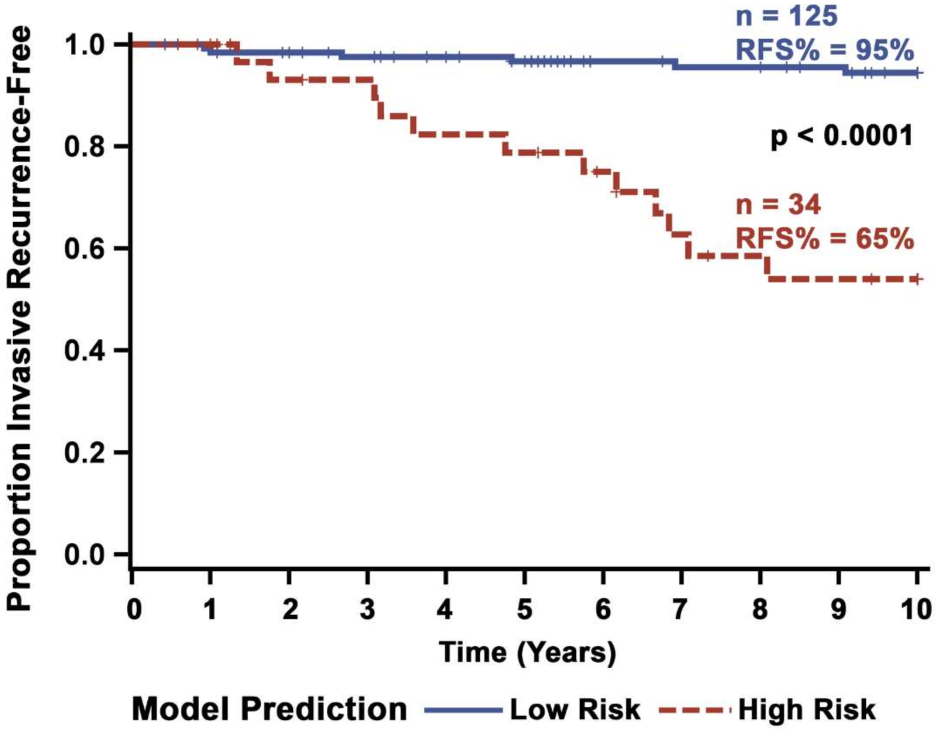

B

Training Cohort Cox Regression for Invasive Recurrence								
Variables		Univariate Analysis			Multivariate Analysis		
		Hazard Ratio	95% Confidence interval	*P*-value	Hazard Ratio	95% Confidence interval	*P*-value
**Recurrence Free Survival**							
Predictive Model	High Risk vs. Low	9.837	3.681 - 26.288	<.0001	8.615	3.145 - 23.597	<0.0001
Comedo Necrosis	Present vs. Absent	0.954	0.370 - 2.462	0.9227	0.54	0.152 - 1.918	0.3406
Size	per mm	0.985	0.959 - 1.012	0.2696	0.3495	0.957 - 1.016	0.8754
Grade	1 vs. 2	-	-	0.9927	-	-	0.9919
Grade	1 vs. 3	-	-	0.9925	-	-	0.9919
Margin	Positive vs. Negative	-	-	0.9929	-	-	0.9946
Age	Per year	1.015	0.958 - 1.075	0.6138	1.038	0.974 - 1.106	0.2469
Radiotherapy	Yes vs. No	0.617	0.178 - 2.136	0.4460	0.745	0.191 - 2.908	0.6719
