# Supplementary material for: A whole slide image-based machine learning approach to predict ductal carcinoma in situ (DCIS) recurrence risk
Source: Breast Cancer Res. 2019 Jul 29;21:83. doi: 10.1186/s13058-019-1165-5 (PMC6664779; doi:10.1186/s13058-019-1165-5)
Supplement: Supplementary file 26 — Supplementary Figure S16. (A) Cross validated Kaplan-Meier curves of patients within the training cohort stratified by the trained recurrence classifier and using only DCIS recurrence as an event. Significance is measured through the log-rank test. (B) Univariate and multivariate Cox regression analysis comparing the influence of common clinicopathological variables alongside the 8-feature recurrence risk prediction model for DCIS recurrence-free survival, on the training set. (PDF 349 kb) [file 13058_2019_1165_MOESM26_ESM.pdf]

A

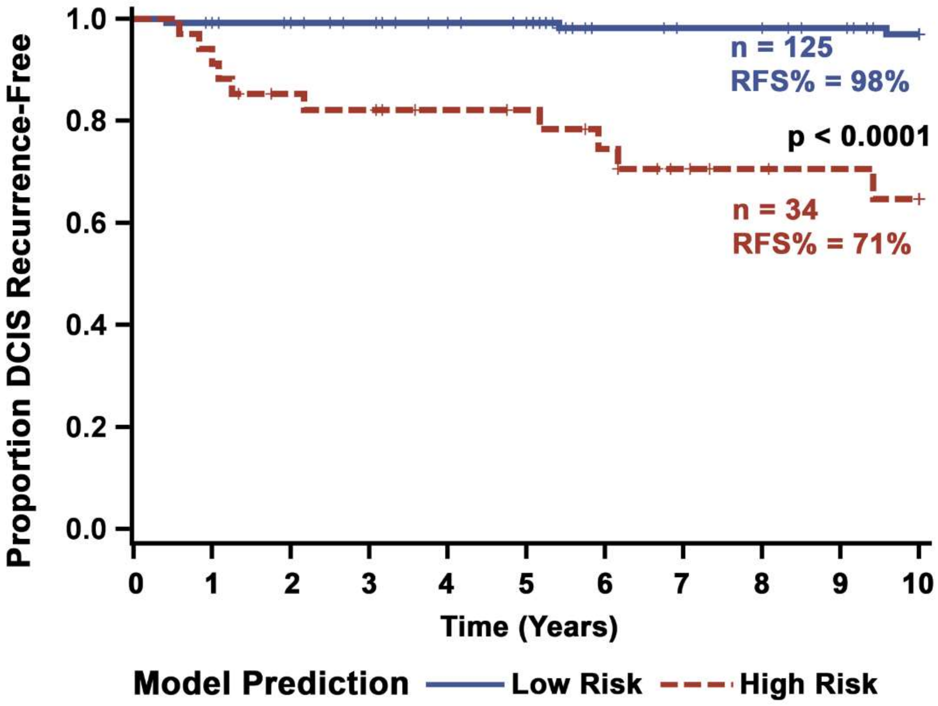

B

Training Cohort Cox Regression for DCIS Recurrence								
		**Univariate Analysis**			**Multivariate Analysis**		
**Variables**		**Hazard Ratio**	**95% Confidence interval**	***P*-value**	**Hazard Ratio**	**95% Confidence interval**	***P*-value**
**Recurrence Free Survival**							
Predictive Model	High Risk vs. Low	15.123	4.139 - 55.252	<.0001	18.181	3.563 - 92.780	0.0005
Comedo Necrosis	Present vs. Absent	0.705	0.237 - 2.099	0.5300	0.916	0.208 - 4.025	0.9074
Size	per mm	0.967	0.922 - 1.013	0.1543	0.971	0.922 - 1.023	0.2721
Grade	1 vs. 2	4.201	0.434 - 40.652	0.2152	1.596	0.139 - 18.299	0.7071
Grade	1 vs. 3	2.283	0.289 - 18.026	0.4336	0.862	0.081 - 9.159	0.9019
Margin	Positive vs. Negative	3.221	0.418 - 24.830	0.2618	1.537	0.133 - 17.772	0.7306
Age	Per year	0.944	0.892 - 1.000	0.0491	0.963	0.902 - 1.029	0.2688
Radiotherapy	Yes vs. No	1.927	0.629 - 5.901	0.2509	2.304	0.625 - 8.497	0.2102
