# Supplementary material for: A whole slide image-based machine learning approach to predict ductal carcinoma in situ (DCIS) recurrence risk
Source: Breast Cancer Res. 2019 Jul 29;21:83. doi: 10.1186/s13058-019-1165-5 (PMC6664779; doi:10.1186/s13058-019-1165-5)
Supplement: Supplementary file 28 — Supplementary Table S9. Univariate cox regression analysis of the impact that continuous metrics can have on both the training (through combining the cross-validation test sets) and validation cohorts. The random survival forest (RSF) was a new model trained with the 8 selected features whereas the RF class probability reflects the continuous score obtained from counting the proportion of trees voting for ‘recurrence’ in the classification model. (PDF 399 kb) [file 13058_2019_1165_MOESM28_ESM.pdf]

Continuous Score Models					
Models			Univariate Analysis		
			Hazard Ratio	95% Confidence interval	*P*-value
**10 Year-Recurrence Free Survival**					
**Trained RSF**	Training Cohort	Per unit score	1.051	1.037 - 1.065	<0.0001
	Validation Cohort	Per unit score	1.051	1.003 - 1.102	0.0358
**RF Class Prob.**	Training Cohort	Per unit score	1.056	1.039 - 1.074	<0.0001
	Validation Cohort	Per unit score	1.029	1.016 - 1.042	<0.0001
