# Supplementary material for: A whole slide image-based machine learning approach to predict ductal carcinoma in situ (DCIS) recurrence risk
Source: Breast Cancer Res. 2019 Jul 29;21:83. doi: 10.1186/s13058-019-1165-5 (PMC6664779; doi:10.1186/s13058-019-1165-5)
Supplement: Supplementary file 31 — Supplementary Fig 20. (A) The Harrell’s c-statistic and 95% confidence interval for the 8-feature model and common clinopathological variables in the validation cohort. (B) The Akaike Information Criterion (AIC) comparing the fit of a null model (no variables), the 8-feature model, and a model composed of the common clinopathological variables (Margins status, necrosis, radiation, age, and size). The lower the AIC value the better the model fits the recurrence data. (PDF 224 kb) [file 13058_2019_1165_MOESM31_ESM.pdf]

A

Variable	Harrell's c-statistic (95% CI)
Predictive Model	**0.69 (0.59 - 0.78)**
Margin Status	0.50 (0.50 - 0.51)
Necrosis	0.53 (0.44 - 0.61)
Radiation	0.55 (0.49 - 0.61)
Age	0.55 (0.44 - 0.67)
Size	0.48 (0.37 - 0.60)

B

Model	Akaike Information Criterion
Null Model	260.53
Predictive Model	**243.00**
Clinopathological Variables	264.64
