# Supplementary material for: A whole slide image-based machine learning approach to predict ductal carcinoma in situ (DCIS) recurrence risk
Source: Breast Cancer Res. 2019 Jul 29;21:83. doi: 10.1186/s13058-019-1165-5 (PMC6664779; doi:10.1186/s13058-019-1165-5)
Supplement: Supplementary file 33 — Supplementary Figure S22. Cross validated Kaplan-Meier curves of patients within the validation cohort, developed by combining the testing sets for a cross validated iteration. (A) Recurrence classifier model used on slides from patients who received adjuvant radiation and (B) Patients who were treated with BCS alone. Significance is measured through the logrank test. (PDF 221 kb) [file 13058_2019_1165_MOESM33_ESM.pdf]

A

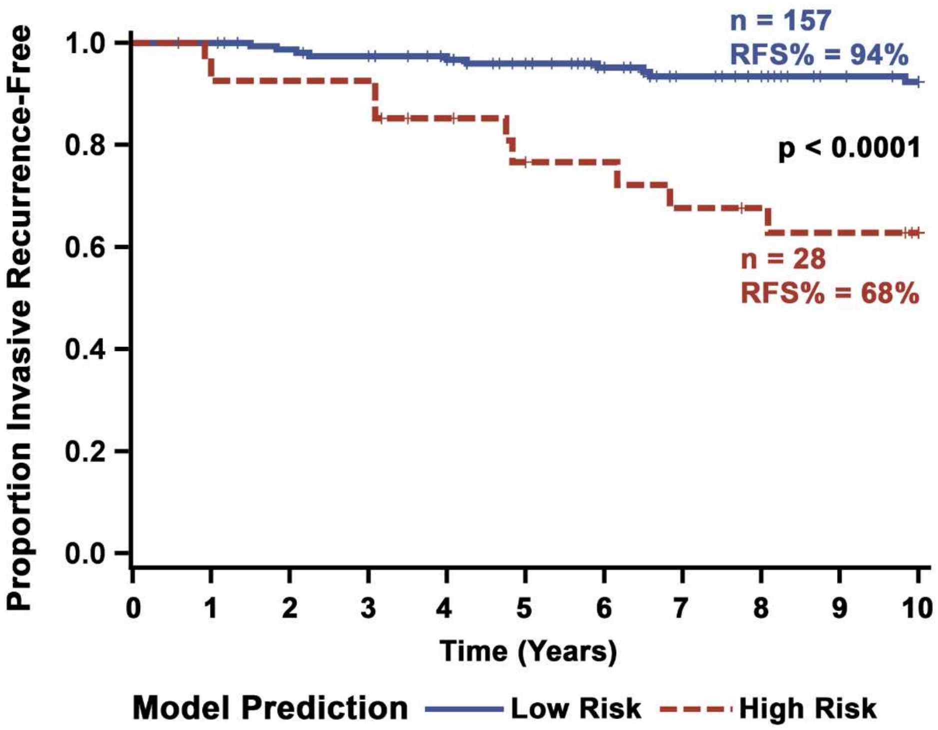

B

Validation Cohort Cox Regression for Invasive Recurrence							
Variables		Univariate Analysis			Multivariate Analysis		
		Hazard Ratio	95% Confidence interval	*P*-value	Hazard Ratio	95% Confidence interval	*P*-value
**Recurrence Free Survival**							
Predictive Model	High Risk vs. Low	5.759	2.339 - 14.178	0.0001	6.287	2.469 - 16.007	0.0001
Comedo Necrosis	Present vs. Absent	0.681	0.245 - 1.893	0.4618	0.948	0.328 - 2.739	0.9219
Size	per mm	1.008	0.990 - 1.027	0.3738	1.012	0.993 - 1.032	0.2035
Margin	Positive vs. Negative	-	-	0.9937	-	-	0.9942
Age	Per year	1.03	0.969 - 1.094	0.3423	1.036	0.975 - 1.101	0.2536
Radiotherapy	Yes vs. No	0.253	0.034 - 1.902	0.1819	0.289	0.037 - 2.246	0.2355
