# Supplementary material for: A whole slide image-based machine learning approach to predict ductal carcinoma in situ (DCIS) recurrence risk
Source: Breast Cancer Res. 2019 Jul 29;21:83. doi: 10.1186/s13058-019-1165-5 (PMC6664779; doi:10.1186/s13058-019-1165-5)
Supplement: Supplementary file 34 — Supplementary Figure S23. (A) Kaplan-Meier curves showing robust stratification of patients in the validation cohort into high-risk of recurrence and low-risk of recurrence subgroups and using only DCIS recurrence as an event. (B) Univariate and multivariate Cox regression analysis comparing the influence of common clinicopathological variables alongside the 8-feature recurrence risk prediction model for DCIS recurrence-free survival, on the validation set. (PDF 325 kb) [file 13058_2019_1165_MOESM34_ESM.pdf]

A

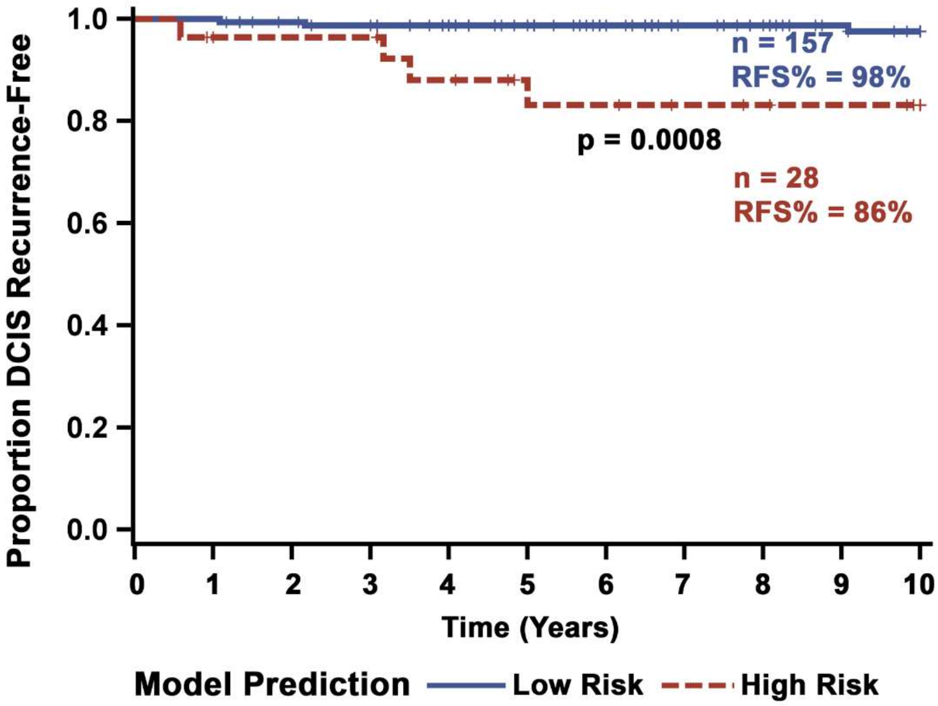

B

Validation Cohort Cox Regression for DCIS Recurrence							
Variables		Univariate Analysis			Multivariate Analysis		
		Hazard Ratio	95% Confidence interval	*P*-value	Hazard Ratio	95% Confidence interval	*P*-value
**Recurrence Free Survival**							
Predictive Model	High Risk vs. Low	8.488	1.898 - 37.962	0.0051	8.289	1.840 - 37.342	0.0059
Comedo Necrosis	Present vs. Absent	1.418	0.171 - 11.791	0.7466	1.688	0.193 - 14.720	0.6358
Size	per mm	0.980	0.931 - 1.033	0.4555	0.985	0.932 - 1.041	0.5883
Margin	Positive vs. Negative	-	-	0.9961	-	-	0.9949
Age	Per year	1.034	0.937 - 1.142	0.5059	1.024	0.922 - 1.136	0.6619
Radiotherapy	Yes vs. No	0.704	0.084 - 5.894	0.7462	0.640	0.073 - 5.621	0.6876
